# Health Care Resource Utilization With Dronedarone Versus Sotalol Following Catheter Ablation in Adults With Atrial Fibrillation

**DOI:** 10.1002/clc.70064

**Published:** 2025-01-15

**Authors:** Emily P. Zeitler, Dara Stein, Ron Preblick, Shaum M. Kabadi, David S. McKindley, Jason Rashkin, Samuel Huse, Nicole Stamas, Michael H. Kim

**Affiliations:** ^1^ Department of Medicine Dartmouth‐Hitchcock Medical Center Lebanon New Hampshire USA; ^2^ Real‐world Evidence Evidera London UK; ^3^ Health Economics and Outcomes Research Sanofi, Bridgewater New Jersey USA; ^4^ Real‐world Evidence Sanofi, Bridgewater New Jersey USA; ^5^ US Medical, Sanofi Bridgewater New Jersey USA; ^6^ Real‐world Evidence Evidera Cambridge Massachusetts USA; ^7^ Real‐world Evidence Evidera Seattle Washington USA; ^8^ Department of Medicine Creighton University School of Medicine and CHI Health Omaha Nebraska USA

**Keywords:** antiarrhythmic drugs, atrial fibrillation, catheter ablation, comparative effectiveness, health care resource utilization, observational cohort, sex analysis (Max 7)

## Abstract

**Background:**

Clinical trials support dronedarone use for atrial fibrillation (AF) following catheter ablation (CA); however, comparative data on health care resource utilization (HCRU) with other antiarrhythmic drugs are lacking.

**Methods:**

Retrospective analysis of Merative MarketScan databases (January 01, 2012−March 31, 2020) comparatively assessed HCRU in US adults with AF who received dronedarone or sotalol post‐CA. Patients with ≥ 12‐months' pre‐CA data were followed from post‐CA index treatment to disenrollment, death, or study end. Sotalol‐treated patients were propensity score‐matched (1:1) with dronedarone‐treated patients. Events/100 patient‐years (PY) were analyzed by univariate generalized‐linear model with Poisson distribution. Cumulative incidence was analyzed over 12 months by Kaplan–Meier methods. Subgroup analyses were conducted by sex and patients new to dronedarone or sotalol during 12 months pre‐CA.

**Results:**

Dronedarone and sotalol cohorts were successfully matched (*n* = 1600 each). Prevalence/100‐PY for all‐cause, cardiovascular (CV)‐related, and atrial tachyarrhythmia (ATA)/AF–related HCRU was lower in dronedarone versus sotalol cohort (all *p* < 0.05). Cumulative incidence for all‐cause, CV‐related, ATA/AF‐related hospitalizations, and pacemaker implantation was lower in dronedarone versus sotalol cohort (all *p* < 0.05). Incidence of all‐cause and CV‐related hospitalizations was lower in dronedarone versus sotalol cohorts in females (*n* = 460) and males (*n* = 1115) (all *p* < 0.05) after rematching. Incidence of ATA/AF‐related hospitalization was lower in males versus females receiving dronedarone. For patients new to dronedarone or sotalol (*n* = 549), HCRU results were generally consistent with primary analyses.

**Conclusion:**

Post‐CA dronedarone, versus sotalol, lowered CV‐related HCRU in all‐comers with AF and in sex subgroups. Findings may contribute to clinical decision making post‐CA in patients with AF.

## Summary

- Clinical management of atrial fibrillation (AF) following catheter ablation (CA) remains challenging, and antiarrhythmic drugs (AADs) play an important role; however, better understanding of tradeoffs when selecting different AADs post‐CA is needed.
- Patient eligibility criteria for dronedarone and sotalol are similar, but clinical and health care outcomes have rarely been compared. Health care resource utilization (HCRU) should be considered alongside clinical outcomes to better inform AAD selection post‐CA.
- Patients with AF prescribed dronedarone post‐CA had generally lower HCRU versus patients prescribed sotalol, largely resulting from reduction in all‐cause and cardiovascular (CV)‐related hospitalizations. The reduction in all‐cause and CV‐related hospitalizations was also noted for patients newly prescribed dronedarone or sotalol, and for female and male patients when analyzed separately.

## Graphical abstract

This real‐world analysis of US adults with atrial fibrillation (AF) who received dronedarone or sotalol post‐catheter ablation (CA) demonstrates that, compared with sotalol, post‐CA dronedarone lowered HCRU in all‐comers with AF and in sex subgroups. These findings may contribute to clinical decision‐making in patients with AF following CA.

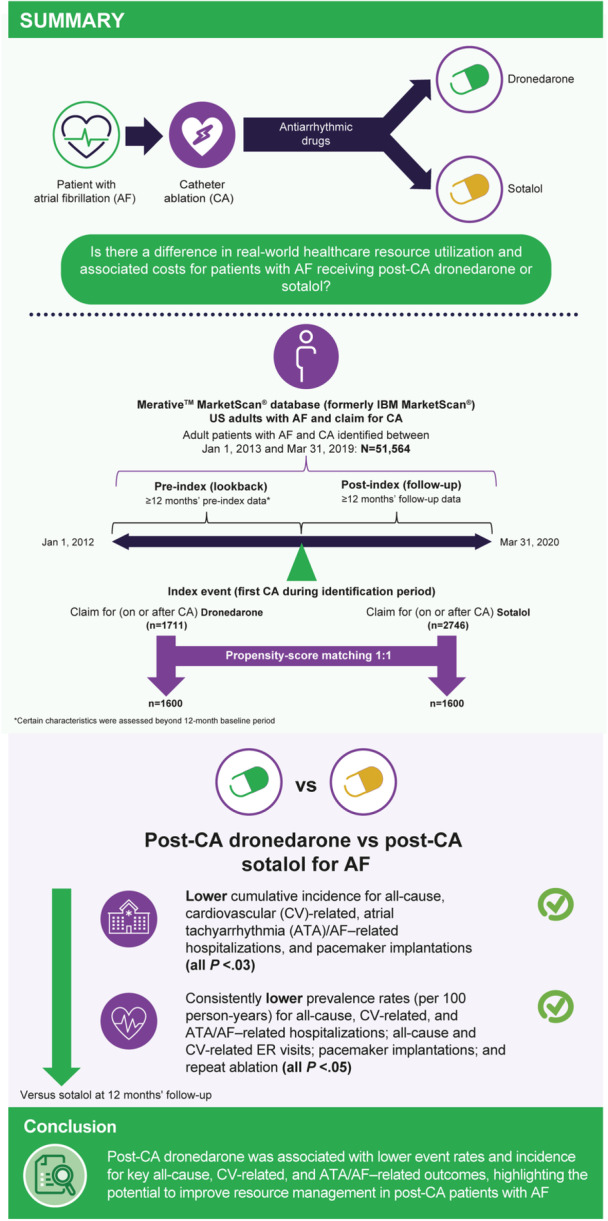

**Abbreviations**
AAD: antiarrhythmic drug
ACE: angiotensin converting enzyme
ACS: acute coronary syndrome
AF: atrial fibrillation
AFL: atrial flutter
ARB: angiotensin receptor blocker
ASD: absolute standard difference
ATA: atrial tachyarrhythmia
CA: catheter ablation
CCB: calcium channel blocker
CCI: Charlson Comorbidity Index
CHD: coronary heart disease
COPD: chronic obstructive pulmonary disease
CPT: Current Procedural Terminology
CV: cardiovascular
DOAC: direct‐acting oral anticoagulant
ECG: electrocardiogram
ER: emergency room
HCRU: health care resource utilization
HF: heart failure
HIPAA: Health Insurance Portability and Accountability Act
LOS: length of stay
MI: myocardial infarction
PAD: peripheral arterial disease
PSM: propensity score matching
PY: patient years
TIA: transient ischemic attack
US: United States
VKA: vitamin K antagonist

## 1 Introduction

Atrial fibrillation (AF) is the most common sustained cardiac arrhythmia [1, 2, 3], with an estimated worldwide prevalence (including atrial flutter [AFL]) of 59.7 million people in 2019 [4], a number which continues to rise [5]. Prevalence estimates in the United States are predicted to increase beyond 12.1 million by 2030 [3, 6], driven by an aging population and an increasing ability to detect AF [2, 3]. The age‐adjusted incidence is reportedly higher in males than females [4, 7, 8]; however, with longer life‐expectancy among females than males, lifetime risk of AF is similar between sexes. The chronic nature of AF and associated comorbidities results in high rates of recurrent hospitalizations and a continuing need for health care services [9, 10]. Indeed, patients with AF are reportedly eight times more likely than patients without AF to have multiple cardiovascular (CV) hospitalizations (4.1% vs. 0.5%) [11]. This contributes to an increasing burden on health care resource utilization (HCRU) [10, 12].

Antiarrhythmic drugs (AADs) can be used alone or in combination with catheter ablation (CA) for suppression of AF [13, 14]. Several AADs are approved for management of AF [13, 14, 15]; however, patient eligibility varies as does the effectiveness, side effects, and logistical considerations such as the variable need for inpatient initiation. In clinical practice, up to 50% of patients continue to receive an AAD after undergoing an ablation procedure [16, 17, 18]; therefore, understanding which AADs are best suited for this purpose is important. The 5A study was a prospective, randomized study which demonstrated that AAD treatment during the first 6 weeks post‐CA reduced the incidence of clinically significant atrial tachyarrhythmias (ATAs) and need for cardioversion/hospitalization for arrhythmia management [19]. Separately, a single‐center observational study of AAD use following first‐line CA, including patients prescribed amiodarone, dronedarone, sotalol, or dofetilide, reported no differences between specific AADs in the risk of early recurrence of AF/AFL/AT whereas patients prescribed amiodarone were the most likely to experience late recurrence [20]. However, limitations of this observational analysis leave questions unanswered about the comparative effectiveness of different AADs after ablation [20].

Although amiodarone is widely prescribed because of its ease of use and relative effectiveness, extra cardiac toxicity is not uncommon [21] and, therefore, guidelines suggest other AADs should be considered first whenever possible [13, 14, 22]. Dronedarone and sotalol are effective in suppressing AF [23, 24, 25], and both can be used post‐CA [20, 26, 27]. Dronedarone and sotalol are recommended for similar patient types, including patients with prior myocardial infarction (MI) or significant structural heart disease, including heart failure (HF) with reduced ejection fraction (left ventricular ejection fraction ≤ 40%), patient types in which Class 1C medications are not recommended [13, 22]. As such, understanding the comparative benefits of dronedarone versus sotalol is of interest, particularly post‐CA where information is relatively sparse.

Using real‐world data derived from claims databases, this study aimed to assess all‐cause HCRU in US adults with AF who received dronedarone or sotalol after CA, overall and in clinically relevant subgroups.

## 2 Methods

### 2.1 Study Design

Data for this retrospective, observational comparative cohort study were derived from the Merative (formally IBM) MarketScan Commercial and Medicare Supplemental databases (January 01, 2012 to March 31, 2020) (Supporting Information S1: Figure S1). Merative MarketScan (MarketScan) databases capture person‐specific clinical utilization, expenditures, and enrollment data across inpatient, outpatient, prescription drug, and other carve‐out services covering > 273 million unique patients [28]. MarketScan Commercial database captures individuals with employer‐sponsored and nongroup private insurance, whereas Medicare Supplemental captures individuals with Medicare‐based plans [28, 29]. Both databases include integrated, patient‐level health care encounters and cost data from inpatient and outpatient settings. MarketScan datasets are Health Insurance Portability and Accountability Act (HIPAA)–compliant and deidentified to adhere with all relevant US regulations and privacy laws.

The baseline period spanned 12 months before, but exclusive of, the date of first CA (index date) during the patient identification period (January 01, 2013 to March 31, 2019), and the follow‐up period spanned from index to the earliest of health plan disenrollment, death, or study end (March 31, 2020). Events on the day of first CA (index) were not collected.

#### 2.1.1 Study Population

Adults (≥ 18 years) with a primary or secondary diagnosis of paroxysmal or persistent AF (ICD‐9 code 427.31 or ICD‐10 codes I48, I48.0, I48.1, I48.91) were eligible for inclusion with a first inpatient or outpatient claim for CA related to AF (index date) during the patient identification period, ≥ 12 months' continuous health‐plan enrollment during the 12‐month baseline period, and an outpatient prescription claim for dronedarone or sotalol, on or after index (Supporting Information S1: Figure S1). Patients with a diagnosis of ventricular arrhythmias, bradycardia, cardioverter defibrillator implantation, pacemaker implantation any time before the date of CA, and/or prescription claim(s) for another AAD (not dronedarone or sotalol) between index and the date of initiation of dronedarone or sotalol post‐CA were excluded.

#### 2.1.2 Variables

All variables and coding definitions were determined a priori. Demographics were based on enrollment details at index. Clinical characteristics and medication prescriptions were analyzed during the 12‐month baseline period. History of AFL was recorded at any time before CA. Charlson Comorbidity Index (CCI) was calculated according to Quan et al. [30] Among patients who contributed one or more comorbidity claims for the same comorbidity but at different severity levels, the more severe comorbidity was selected. CHA_2_DS_2_‐VASc scores were defined according to Lip et al. [31] Patients were considered recently treated if in possession of a medication on the day before or within 30 days before index [32]. Patients were assumed to be in possession of the prescription starting on the fill date and ending on the run‐out date (fill date plus days supplied).

#### 2.1.3 Propensity Score Matching (PSM)

PSM was carried out to match sotalol‐treated patients 1:1 with dronedarone‐treated patients, applying a greedy matching algorithm without replacement, and adjusting for variables shown in Supporting Information S1: Table S1. As baseline HCRU can be an important predictor of post‐index HCRU, baseline HCRU was part of the matching scheme. Patients receiving dronedarone were ordered and sequentially matched to the nearest unmatched sotalol‐treated patient. If one or more unmatched sotalol‐treated patients were linked, the sotalol‐treated patient was selected at random. Once matched, they were no longer available (without replacement). Characteristics left unbalanced following PSM (absolute standardized difference [ASD] > 0.1) were adjusted for as covariates.

Subgroups were analyzed and underwent PSM using the same approach, including females, males, and new users of dronedarone or sotalol at time of CA (“new to index AAD”), defined as patients who did not receive the same first post‐CA AAD (i.e., dronedarone or sotalol, depending on cohort) during the 12‐month baseline period. Presence of ATA/AF–related hospitalization at baseline was added to the matching scheme for males as this remained unbalanced after initial PSM.

#### 2.1.4 HCRU

##### 2.1.4.1 Prevalence Rates

After PSM, prevalence rates for all‐cause, CV‐related, and ATA/AF‐related HCRU were calculated as the total aggregate number of events during follow‐up reported per 100‐patient‐years (PY), where patients were considered at‐risk from date of index CA to end of follow‐up (database disenrollment, death, or study end). Prevalence rates were calculated for individual components of HCRU including hospitalizations, emergency room (ER) visits, any outpatient office visits and other outpatient services, pacemaker implantation, and repeat CA. The components of outpatient office visits and other outpatient services are provided in Supporting Information S1: Table S2. HCRU rates included the initial event plus any subsequent clinical events. CV‐related HCRU was defined as hospitalizations, ER visits, outpatient visits, and other outpatient services with an ICD‐9/ICD‐10 diagnosis code in the primary position related to AF, ATA, ischemic stroke/transient ischemic attack, MI, ventricular arrhythmias, bradycardia, or HF; prescription for CV‐related drugs; or hospitalization for acute coronary syndrome/MI, HF, or ischemic stroke (Supporting Information S1: Table S2). ATA/AF‐related hospitalizations and ER visits were used as surrogate parameters to indirectly capture AF recurrence, cardioversions, and other AF‐related procedures within the claims data. AF/ATA‐related HCRU was defined as primary ICD‐9/10 diagnosis code of AF, ATA, or prescription for AF‐related medications (other than index dronedarone or sotalol), organized into hospitalizations, ER visits, outpatient office visits, other outpatient services, and AF‐related procedures (cardioversion and/or repeat CA). AF‐related medications included AADs (dronedarone, sotalol, amiodarone, flecainide, propafenone, and dofetilide), beta‐blockers, calcium channel blockers (CCBs) (both nondihydropyridine [NDHP] and dihydropyridine [DHP]), digoxin, direct oral anticoagulants (DOACs), vitamin K antagonist/warfarin, and P2Y12 inhibitors (antiplatelets) (see Supporting Information S1: Table S2).

##### 2.1.4.2 Cumulative Incidence

Incidence rates included the initial event only. In the PSM cohorts, cumulative incidence rates were estimated using a Kaplan–Meier analysis with separate survival curves for dronedarone and sotalol cohorts. Kaplan–Meier methods were used to estimate cumulative incidence until each first CV‐related event of interest; this was depicted using survival curves. Follow‐up time for patients who did not experience a particular event was censored at the earliest of health plan disenrollment, death, or study end. The same analysis was used for PSM subgroups: by sex (female, male) and for new users of dronedarone or sotalol at the time of CA (new to index AAD).

Kaplan–Meier methods were also used to estimate the cumulative incidence for duration on AAD therapy following CA and time‐to‐first switch from study drug (dronedarone or sotalol) to another AAD following CA.

#### 2.1.5 Statistical Analysis

Continuous variables were summarized using means, standard deviations, medians, and interquartile ranges. Categorical variables were summarized using counts with percentages. No imputations were made for missing variables; for the purpose of this analysis, missing variables were reported as missing or unknown.

The primary objective was to assess HCRU prevalence rates between patients treated with dronedarone versus sotalol. Event rates were compared using univariate generalized linear models with Poisson distribution. For incidence time‐to‐event Kaplan–Meier analysis, *p*‐values were calculated using log‐rank test between cumulative incidence of event between cohorts at 12 months post‐CA. Statistical significance was set at a two‐sided alpha of 0.05. The same analyses were used for PSM subgroups: by sex (female, male) and patients new to index AAD.

## 3 Results

### 3.1 Populations and Subgroups

**Figure 1:**
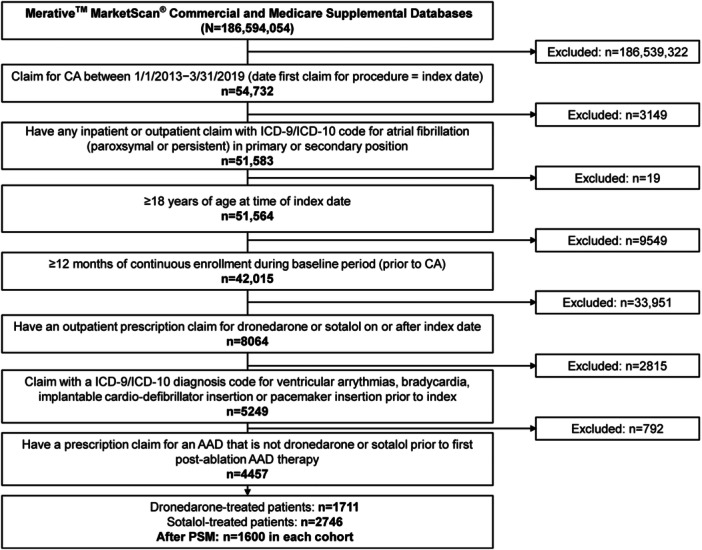
Study attrition for overall cohort. AAD, antiarrhythmic drug; CA, catheter ablation; ICD‐9/ICD‐10, International Classification of Diseases 9th/10th Revision; PSM, propensity score matching.

After applying eligibility criteria, 4457 adults with AF prescribed dronedarone (*n* = 1711) or sotalol (*n* = 2746) were identified (Figure 1). After PSM, the dronedarone and sotalol cohorts (*n* = 1600 each) were well‐matched across demographic and baseline clinical characteristics (Table 1; Supporting Information S1: Figure S2). However, urban residency and number of ECGs during the baseline period remained unbalanced (ASD > 0.1; Table 1).

**Table 1:** Baseline characteristics, medication use, and HCRU before and after PSM.

Characteristics mean ± SD or *n* (%)	Before PSM		ASD	After PSM		ASD^a^
	Dronedarone (*n* = 1711)	Sotalol (*n* = 2746)		Dronedarone (*n* = 1600)	Sotalol (*n* = 1600)	
Age at index^b^, years	61.3 ± 9.4	60.9 ± 8.9	0.049	61.3 ± 9.4	61.2 ± 9.1	0.009
Sex^b^						
Female	511 (29.9)	806 (29.4)	0.011	479 (29.9)	488 (30.5)	0.012
Male	1200 (70.1)	1940 (70.6)		1121 (70.1)	1112 (69.5)	
Region^b^						
Northeast	454 (26.5)	469 (17.1)	0.231	390 (24.4)	360 (22.5)	0.044
North Central	314 (18.4)	606 (22.1)	0.093	306 (19.1%)	316 (19.8)	0.016
South	690 (40.3)	1195 (43.5)	0.065	659 (41.2)	669 (41.8)	0.013
West	240 (14.0)	460 (16.8)	0.076	235 (14.7)	245 (15.3)	0.018
Unknown	13 (0.8)	16 (0.6)	0.022	10 (0.6)	10 (0.6)	0.000
Urban residency	1382 (80.8)	2106 (76.7)	0.100	1296 (81.0)	1228 (76.8)	0.104
Medicare supplemental^b^	1195 (69.8)	2034 (74.1)	0.094	1128 (70.5)	1138 (71.1)	0.014
Follow‐up, months	29.0 ± 20.6	27.8 ± 20.5	0.061	28.9 ± 20.5	28.1 ± 20.5	0.041
Selected comorbidities^c^						
Hypertension^b^	1184 (69.2)	2109 (76.8)	0.172	1137 (71.1)	1151 (71.9)	0.019
CHD^b^	649 (37.9)	1096 (39.9)	0.041	611 (38.2)	618 (38.6)	0.009
Diabetes^b^	313 (18.3)	660 (24.0)	0.141	295 (18.4)	310 (19.4)	0.024
COPD^b^	256 (15.0)	372 (13.5)	0.041	85 (5.3)	95 (5.9)	0.027
Heart failure^b^	205 (12.0)	432 (15.7)	0.109	200 (12.5)	211 (13.2)	0.021
PAD^b^	132 (7.7)	212 (7.7)	0.000	119 (7.4)	126 (7.9)	0.017
Ischemic stroke^b^	62 (3.6)	78 (2.8)	0.044	55 (3.4)	49 (3.1)	0.021
MI^b^	49 (2.9)	97 (3.5)	0.038	48 (3.0)	54 (3.4)	0.021
Vascular disease^b^	36 (2.1)	56 (2.0)	0.005	32 (2.0)	36 (2.3)	0.017
Venous thromboembolism^b^	27 (1.6)	58 (2.1)	0.040	26 (1.6)	32 (2.0)	0.028
History of AFL^b^, ^d^	584 (34.1)	986 (35.9)	0.037	544 (34.0)	547 (34.2)	0.004
CCI^b^, ^e^	0.7 ± 1.2	0.9 ± 1.3	0.111	0.8 ± 1.3	0.8 ± 1.2	0.036
CHA_2_DS_2_‐VASc score^b^, ^e^	1.8 ± 1.4	1.9 ± 1.3	0.080	1.8 ± 1.4	1.9 ± 1.3	0.020
Baseline procedures						
ECG^b^	1694 (99.0)	2704 (98.5)	0.048	1583 (98.9)	1588 (99.3)	0.033
Number of ECG	5.6 ± 3.6	6.2 ± 3.9	0.146	5.7 ± 3.7	6.1 ± 3.9	0.121
Cardioversion^b^	586 (34.2)	1206 (43.9)	0.199	571 (35.7)	593 (37.1)	0.066
Index CA setting						
Inpatient	127 (7.4)	369 (13.4)	0.198	120 (7.5)	228 (14.3)	0.218
Outpatient	1584 (92.6)	2377 (86.6)	0.198	1480 (92.5)	1372 (85.8)	0.218
Time to first post‐CA AAD therapy^b^						
< 90 days	1530 (89.4)	2444 (89.0)	0.014	1436 (89.8)	1418 (88.6)	0.036
≥ 90 days	181 (10.6)	302 (11.0)	0.014	164 (10.3)	182 (11.4)	0.036
First postablation AAD therapy duration, months (median [range])	3 (0–78)	4 (0–84)	0.4356	3 (0–78)	4 (0–83)	0.4343
Baseline medication use^b^						
Any AAD before baseline	1292 (75.5)	2312 (84.2)	0.218	1265 (79.1)	1290 (80.6)	0.039
Any DOAC	1227 (71.7)	1909 (69.5)	0.048	1163 (72.7)	1168 (73.0)	0.007
Beta‐blockers	1201 (70.2)	1611 (58.7)	0.243	1125 (70.3)	1126 (70.4)	0.001
CCBs (DHP, NDHP)	659 (38.5)	1086 (39.5)	0.021	631 (39.4)	653 (40.8)	0.028
ACE inhibitors	428 (25.0)	929 (33.8)	0.194	419 (26.2)	437 (27.3)	0.025
VKA/warfarin	351 (20.5)	680 (24.8)	0.102	342 (21.4)	340 (21.3)	0.003
ARBs	327 (19.1)	522 (19.0)	0.003	314 (19.6)	307 (19.2)	0.011
Loop diuretics	207 (12.1)	461 (16.8)	0.134	203 (12.7)	223 (13.9)	0.037
Digoxin	170 (9.9)	351 (12.8)	0.090	164 (10.3)	167 (10.4)	0.006
P2Y12 inhibitors	135 (7.9)	202 (7.4)	0.049	167 (10.4)	162 (10.1)	0.010
Aldosterone antagonists	50 (2.9)	123 (4.5)	0.083	50 (3.1)	42 (2.6)	0.030
Time from CA to first AAD Rx^b^	59.8 ± 183.7	56.3 ± 155.1	0.021	59.2 ± 182.8	59.3 ± 169.8	0.000
Baseline HCRU						
Outpatient visits^b^	19.8 ± 15.6	19.4 ± 14.5	0.027	19.8 ± 15.3	20.1 ± 15.7	0.023
ATA/AF‐related outpatient visits^b^	5.2 ± 3.9	5.5 ± 4.2	0.054	5.3 ± 4.0	5.3 ± 4.0	0.006
CV‐related outpatient visits^b^	5.3 ± 4.0	5.6 ± 4.3	0.058	5.4 ± 4.1	5.4 ± 4.1	0.005
≥ 1 inpatient hospitalization^b^	486 (28.4)	988 (36.0)	0.163	478 (29.9)	512 (32.0)	0.046
≥ 1 ER visit^b^	611 (35.7)	964 (35.1)	0.013	578 (36.1)	579 (36.2)	0.001

Abbreviations: AAD, antiarrhythmic drug; ACE, angiotensin converting enzyme; AFL, atrial flutter; ARB, angiotensin receptor blocker; ASD, absolute standard difference; ATA/AF, atrial tachyarrhythmia/atrial fibrillation; CA, catheter ablation; CCB, calcium channel blocker; CCI, Charlson Comorbidity Index; CHD, coronary heart disease; COPD, chronic obstructive pulmonary disease; CV, cardiovascular; DHP, dihydropyridine; DOAC, direct‐acting oral anticoagulant; ECG, electrocardiogram; ER, emergency room; HCRU, health care resource utilization; MI, myocardial infarction; NDHP, nondihydropyridine; PAD, peripheral arterial disease; PSM, propensity score matching; Rx, prescription; SD, standard deviation; VKA, vitamin K antagonist.

^a^ A covariate was considered balanced if ASD between cohorts was ≤ 0.1 (10%). Characteristics left unbalanced following PSM (ASD > 0.1) were adjusted as covariates.

^b^ Included in PSM (Supporting Information S1: Table S1).

^c^ Selected comorbidities recorded in 12‐month baseline period.

^d^ At any time before index CA.

^e^ CHA_2_DS_2_‐VASc and CCI calculated during baseline period were both continuous.

Following separate PSM of female and male patients from the overall cohort, there were 460 female and 1115 male patients in each cohort (sotalol and dronedarone). Baseline characteristics were generally well‐balanced for females (Supporting Information S1: Table S3A) and males (Supporting Information S1: Table S3B). Following PSM of patients new to index AAD (dronedarone or sotalol), there were 549 patients in each cohort (Supporting Information S1: Table S4). Patient characteristics were generally similar in this subgroup, although slightly more patients in the dronedarone cohort were based in urban areas (84.3% vs. 79.4% sotalol; ASD 0.13), as seen in the overall cohort. In patients new to index AAD (dronedarone or sotalol), a higher proportion in the dronedarone cohort had undergone CA as an outpatient versus in the sotalol cohort (Supporting Information S1: Table S4). This was also noted in the overall cohort (Table 1) and when analyzed by sex (Supporting Information S1: Table S3).

### 3.2 Treatment Discontinuation and AAD Switching: Overall Cohort

**Figure 2:**
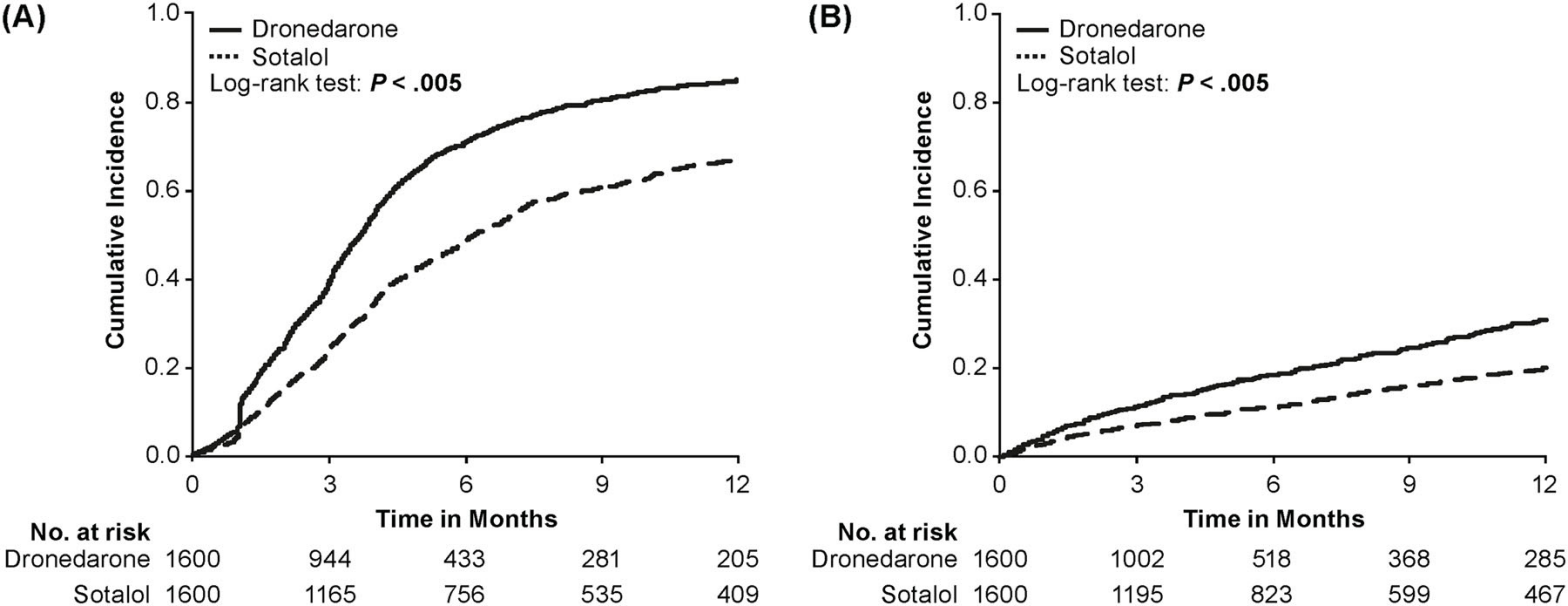
Cumulative incidence for (A) time‐to‐discontinuation from index AAD, and (B) time‐to‐switch from index AAD to another AAD for patients in dronedarone or sotalol cohorts after PSM over 12‐month follow‐up. *p*‐value comparing survival times between dronedarone and sotalol cohorts for each outcome. AAD, antiarrhythmic drug; PSM, propensity score matching.

Median treatment duration was 4 months in the sotalol cohort and 3 months in the dronedarone cohort (Table 1). There was a significantly higher cumulative incidence of AAD discontinuation over 12‐month follow‐up among the dronedarone versus sotalol cohort following CA (Figure 2A); the proportion of patients discontinuing at any time during follow‐up was 88.1% (1410/1600) in the dronedarone cohort and 76.2% (1219/1600) in the sotalol cohort. In total, 1026/1600 dronedarone‐treated patients (64.1%) and 960/1600 sotalol‐treated patients (60.0%) used no further AAD following discontinuation (Supporting Information S1: Table S5). Dronedarone‐treated patients had a significantly higher incidence of switching to another AAD versus sotalol‐treated patients (Figure 2B). In total, 315/1600 dronedarone‐treated patients (19.7%) and 241/1600 sotalol‐treated patients (15.1%) switched to a different AAD after discontinuation (defined as > 30‐day gap between run‐out date and fill date of next AAD prescription). A substantial proportion of patients restarted the same AAD after discontinuation (dronedarone, 13.4% [214/1600]; sotalol, 21.9% [350/1600]) (Supporting Information S1: Table S5).

### 3.3 HCRU: Overall Cohort

For multiple HCRU measures, prevalence rates (events per 100 PY) were significantly lower in the dronedarone versus sotalol cohort, including all‐cause (24.0 vs. 27.4), CV‐related (8.4 vs. 11.3), and ATA/AF‐related hospitalizations (6.7 vs. 9.7), all‐cause (53.1 vs. 59.5) and CV‐related ER visits (8.5 vs. 10.0), repeat CA (14.8 vs. 16.7), and pacemaker implantation (1.5 vs. 2.6) (Table 2). ATA/AF‐related outpatient office visits (365.1 vs. 374.6) and other outpatient services (234.8 vs. 243.0) were also significantly lower in the dronedarone versus sotalol cohort (Table 2).

**Table 2:** Prevalence rates for all‐cause and CV‐related HCRU after PSM.

Outcomes			Rate per 100‐PY (95% CI)			Rate per 100‐PY (95% CI)	*p*‐value
	Patients with event, *n* (%)	Total events	Dronedarone (*n* = 1600)^a^	Patients with event, *n* (%)	Total events	Sotalol (*n* = 1600)^a^	
All‐cause							
Hospitalization	514 (32.1)	925	24.0 (22.4–25.5)	574 (35.9)	1027	27.4 (25.7–29.1)	0.003
ER visit	656 (41.0)	2051	53.1 (50.8–55.4)	650 (40.6)	2232	59.5 (57.1–62.0)	< 0.001
Outpatient office visit	1572 (98.3)	73,408	1901.0 (1887.3–1914.8)	1582 (98.9)	71 718	1912.6 (1898.6–1926.6)	0.249
Other outpatient services	1582 (98.9)	44,023	1140.1 (1129.4–1150.7)	1570 (98.1)	44 415	1184.5 (1173.5–1195.5)	< 0.001
CV‐related							
Hospitalization	253 (15.8)	324	8.4 (7.5–9.3)	321 (20.1)	424	11.3 (10.2–12.4)	< 0.001
ER visit	167 (10.4)	328	8.5 (7.6–9.4)	208 (13.0)	375	10.0 (9.0–11.0)	0.031
Outpatient office visit	1503 (93.9)	14,657	379.6 (373.4–385.7)	1525 (95.3)	14 659	390.9 (384.6–397.3)	0.012
Other outpatient services	1423 (88.9)	10,188	263.8 (258.7–269.0)	1414 (88.4)	9970	265.9 (260.7–271.1)	0.584
Pacemaker implantation	45 (2.8)	59	1.5 (1.1–1.9)	70 (4.4)	99	2.6 (2.1–3.2)	0.001
ATA/AF‐related							
Hospitalization	208 (13.0)	260	6.7 (5.9–7.6)	282 (17.6)	365	9.7 (8.7–10.7)	< 0.001
ER visit	146 (9.1)	290	7.5 (6.7–8.4)	176 (11.0)	320	8.5 (7.6–9.5)	0.115
Outpatient office visit	1498 (93.6)	14099	365.1 (359.1–371.1)	1523 (95.2)	14,048	374.6 (368.4–380.8)	0.031
Other outpatient services	1411 (88.2)	9068	234.8 (230.0–239.7)	1397 (87.3)	9113	243.0 (238.0–248.0)	0.021
Repeat CA	363 (22.7)	573	14.8 (13.6–16.1)	385 (24.1)	625	16.7 (15.4–18.0)	0.045

Abbreviations: ATA/AF, atrial tachyarrhythmia/atrial fibrillation; CA, catheter ablation; CI, confidence interval; CV, cardiovascular; ER, emergency room; HCRU, health care resource utilization; PSM, propensity score matching; PY, patient‐year.

^a^ Variables used in PSM are in Supporting Information S1: Table S1. HCRU recorded at any time after index.

**Figure 3:**
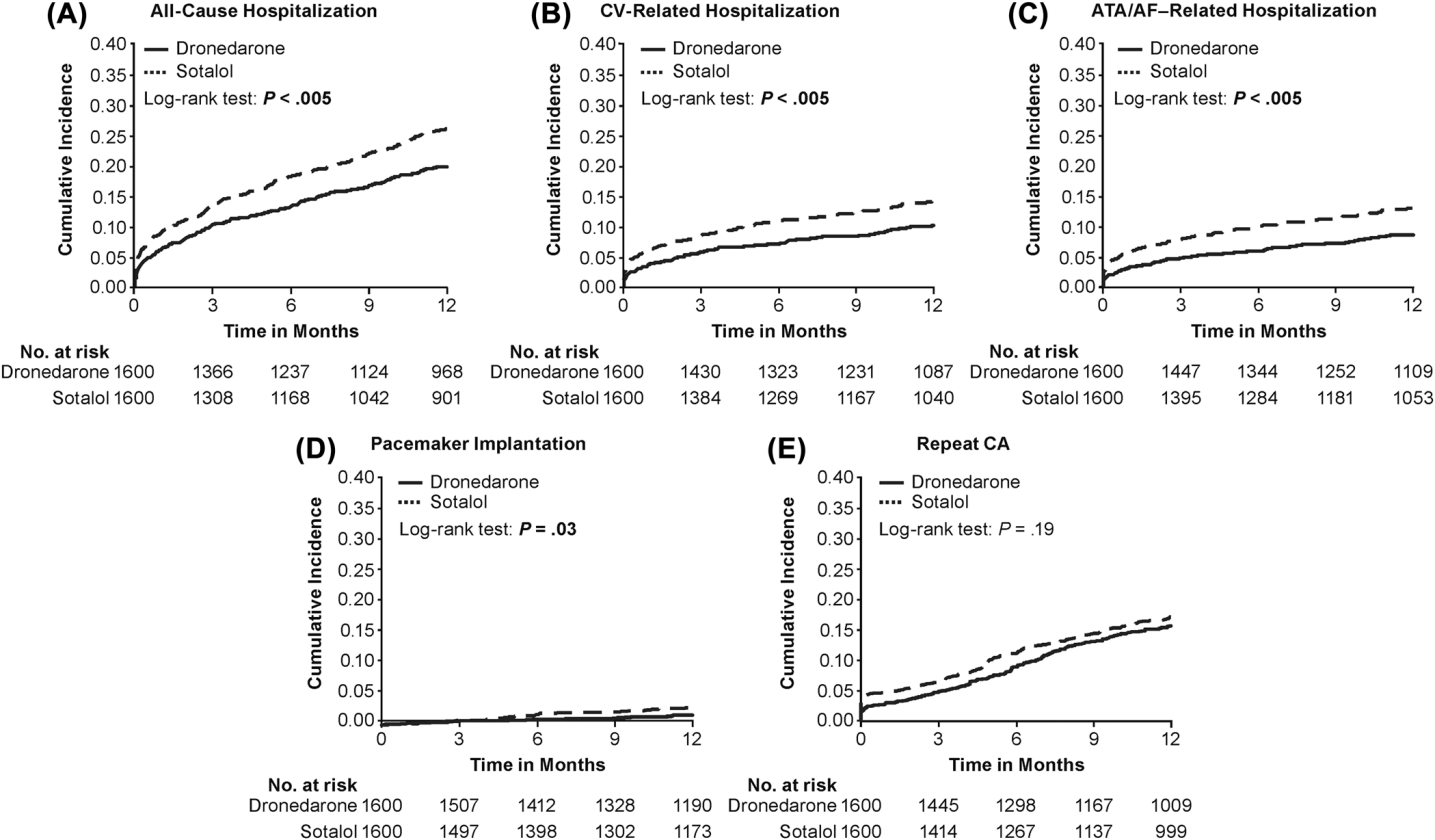
Cumulative incidence rate for (A) all‐cause hospitalization, (B) CV‐related hospitalization, (C) ATA/AF–related hospitalization, (D) pacemaker implantation, (E) repeat CA for patients within the dronedarone or sotalol cohorts after PSM over 12‐months follow‐up. ATA/AF, atrial tachyarrhythmia/atrial fibrillation; CA, catheter ablation; CV, cardiovascular; PSM, propensity score matching. *p*‐value comparing survival times between dronedarone and sotalol cohorts for each outcome.

Cumulative incidence rates for all‐cause, CV‐related, and ATA/AF‐related hospitalizations and pacemaker implantation were significantly lower in the dronedarone versus sotalol cohort over 12‐months' follow‐up (Figure 3). There was no significant difference in cumulative incidence for repeat CA over 12‐months’ follow‐up.

### 3.4 HCRU: Sex Subgroups

#### 3.4.1 Females

In the female subgroup (*n* = 460 each cohort), post‐CA prevalence rates per 100 PY for all‐cause ER visits (57.4 vs. 75.3), outpatient office visits (2156.6 vs. 2221.6), other outpatient services (1220.5 vs. 1360.4), CV‐related hospitalizations (10.3 vs. 13.7) CV‐related ER visits (10.3 vs. 14.1), ATA/AF‐related hospitalizations (8.7 vs. 11.9), and all‐cause ER visits (9.1 vs. 12.4) were significantly lower in the dronedarone versus sotalol cohort, respectively (Supporting Information S1: Table S6). Unlike the overall cohort, there was no difference in prevalence for repeat CA (17.5 vs. 18.0) with nearly overlapping 95% confidence intervals.

Cumulative incidence rates for all‐cause and CV‐related hospitalizations were significantly lower in the dronedarone versus sotalol cohort (Supporting Information S1: Figure S3A) over 12‐months' follow‐up. However, no difference in cumulative incidence rates for ATA/AF‐related hospitalizations or repeat CA was seen over 12‐months' follow‐up.

#### 3.4.2 Males

In the male subgroup (*n* = 1115 for each cohort), post‐CA prevalence (events per 100 PY) for all‐cause outpatient office visits (1786.7 vs. 1711.5), other outpatient services (1087.7 vs. 1107.9), and CV‐related (7.5 vs. 9.7) and ATA/AF‐related hospitalizations (5.8 vs. 8.1) was significantly lower in the dronedarone versus sotalol cohort (Supporting Information S1: Table S6). Prevalence of all‐cause ER visits and CV‐related and ATA/AF‐related ER visits were similar among males in the dronedarone versus sotalol cohorts. Similar to the female subgroup, there was no difference in the prevalence of repeat CA (13.4 vs. 15.0) between cohorts (Supporting Information S1: Table S6).

Cumulative incidence rates over 12‐months' follow‐up in the male subgroup mirrored those in the overall cohort for incidence of all‐cause, CV‐related, and ATA/AF‐related hospitalizations, and were significantly lower for male patients in the dronedarone versus sotalol cohort over 12‐months' follow‐up (Supporting Information S1: Figure S3B). However, among males, there was no significant difference in the cumulative incidence rate for repeat CA over 12‐months' follow‐up.

### 3.5 HCRU: New to Index AAD

For multiple HCRU measures, prevalence rates (events per 100 PY) were significantly lower among the dronedarone versus sotalol cohorts, including for all‐cause (23.4 vs. 32.5), CV‐related (9.6 vs. 15.4), and ATA/AF‐related hospitalizations (7.3 vs. 14.1) all‐cause (51.7 vs. 64.0) and CV‐related ER visits (9.8 vs. 13.2), and pacemaker implantation (1.5 vs. 3.7) in the new to index AAD subgroup (Supporting Information S1: Table S7).

In addition, cumulative incidence rates over 12‐months' follow‐up for CV‐related hospitalizations (Supporting Information S1: Figure S4A), ATA/AF‐related hospitalizations (Supporting Information S1: Figure S4B), and pacemaker implantation (Supporting Information S1: Figure S4C) were significantly lower for the dronedarone versus sotalol cohort, which mirrored HCRU seen in the overall cohort. In the new to index AAD subgroup, repeat CA was also lower in the dronedarone versus sotalol cohort (Supporting Information S1: Figure S4D).

## 4 Discussion

In this comparative cohort analysis of post‐CA patients with AF from the US eligible for both sotalol and dronedarone, dronedarone treatment was generally associated with lower HCRU compared with sotalol. Furthermore, cumulative incidence was significantly lower with dronedarone versus sotalol treatment for all‐cause, CV‐related, and ATA/AF‐related HCRU, and for time to pacemaker implantation. Over 12 months post‐CA, there was no difference in cumulative incidence of repeat CA between cohorts. In sex subgroups and patients new to dronedarone or sotalol therapy at index, results were generally similar to the overall cohort and favored reduction in all‐cause and CV‐related hospitalizations in the dronedarone cohort. Collectively, these findings reflect generally lower HCRU, largely resulting from a reduction in all‐cause and CV‐related hospitalizations associated with management of AF in the challenging post‐CA space with dronedarone versus sotalol.

We specifically chose a comparison of sotalol and dronedarone since these AADs were similarly recommended in patients with structural heart disease at the time of this analysis [13, 22]. The updated ACC/AHA/ACCP/HRS guidelines for the diagnosis and management of AF reflect a stronger recommendation for dronedarone over sotalol due to greater likelihood of benefit over harm in patients with AF and without HF with reduced ejection fraction [13]. This change, however, does not take into account differences in HCRU. Observations from the present study provide further support for this change: following matching, dronedarone was associated with lower HCRU compared with sotalol, as assessed by reduction in event rate prevalence and cumulative incidence for all‐cause hospitalization, CV‐related hospitalizations, and ATA/AF‐related hospitalizations. In addition, time to pacemaker implantation was significantly lower in dronedarone versus sotalol cohorts, which may be predicted by the stronger bradycardia‐inducing effects of sotalol [27]. There was no difference between cohorts for the cumulative incidence of repeat CA. A greater number of patients discontinued the AAD or switched AADs in the dronedarone cohort; specific reasons behind discontinuation or AAD switching are not recorded within these claims data, but could be due to effectiveness or safety, severity of illness, or clinical history or comorbidities [33, 34]. The majority of patients either discontinued from AADs completely (dronedarone: 64%; sotalol: 60%) within 12 months, in line with studies suggesting that up to 50% of patients continue to receive AAD after undergoing an ablation procedure [16, 17, 18], or restarted their index AAD (dronedarone: 13%, sotalol: 22%) [19, 20]. A reduction in HCRU among dronedarone‐treated patients with AF post‐CA has been reported versus patients treated with placebo in the ATHENA study [26, 35, 36], including a 26% reduced risk of CV‐related hospitalizations [24, 36]. Our observations are aligned with an earlier comparative cohort study of adults with AF post‐CA (2013‐2017) demonstrating that patients treated with dronedarone after CA had lower absolute risk of CV hospitalization at 6 and 12 months compared with patients treated with sotalol, predominantly attributed to lower hazard of ATA‐ and HF‐related hospitalizations [27]. Our study extends this prior work by investigating event rates and incidence of HCRU in sex subgroups (female and male) and in patients new to AAD treatment, as well as exploring important additional HCRU endpoints (e.g., ER visits) in these subgroups as well as in the post‐CA cohort as a whole.

Differences in risk factors, comorbidities, clinical presentation, and CV HCRU have been documented between the sexes in patients with AF [37, 38, 39]; however, the impact of sex on HCRU specifically among post‐CA patients treated with AADs remains poorly understood. We noted a reduction in HCRU in both female and male patients with AF post‐CA with dronedarone compared with sotalol in the subgroup analyses, consistent with previous data of dronedarone given post‐CA [27, 35]. When evaluated in sex subgroups, the incidence of all‐cause and CV‐related hospitalizations was lower in the dronedarone cohorts, as seen in the overall cohort. Both female and male cohorts receiving dronedarone had a significantly lower HCRU event rate compared with those receiving sotalol as seen with the overall cohort. Interestingly, female patients treated with dronedarone had a significantly lower rate of ER visits, including CV‐related and ATA/AF‐related ER visits, compared with patients receiving sotalol, whereas there was no difference in the rate of ER visits between the dronedarone and sotalol cohorts in the male subgroup analysis. The reasons behind these observations are unknown, although it should be noted that female and male subgroup analyses were developed independently, and no statistical comparisons were performed between males and females since sex is not a modifiable risk factor. Future, prospective work is needed to further untangle the relationship between sex and optimal post‐CA management of AF. In addition, further research is needed to assess mortality risk associated with dronedarone versus sotalol use post‐CA in female and male patients and in those new to their AAD treatment. For now, although primarily descriptive, our findings can contribute to clinical decision‐making around post‐CA management and suggest benefits in reducing HCRU associated with dronedarone over sotalol.

### 4.1 Strengths and Limitations

This study was conducted on the basis of an “intent‐to‐treat approach” using a comparative cohort design and trial emulation [40], and it followed associations between upstream decision‐making with downstream HCRU. The breadth of coverage allowed robust conclusions to be drawn about HCRU among the overall population and in selected subgroups. In addition, the comparative cohort design facilitated similarity in measured patient characteristics between cohorts, and post‐PSM was used to reduce the potential for unmeasured confounding, and made it possible to improve the clinical relevance of the research question [41]. Although patients with AF prescribed dronedarone or sotalol after CA included in this analysis had similar characteristics, some variability was expected. Various methods are available to address this kind of confounding; in this case PSM was employed before calculation of HCRU prevalence rates and cumulative incidence. A range of different ways can be used to balance distribution of scores following estimation of propensity scores, and in turn confounding factors, across cohorts being compared [42]. This includes propensity score adjustment, matching, stratification, and weighting. Of the different matching techniques, PSM was chosen since the baseline characteristics were quite unbalanced between the two cohorts and the sample size was large enough to accommodate 1:1 matching [43, 44].

Using the Merative MarketScan Commercial and Medicare Supplemental databases allowed us to investigate a large, geographically diverse sample of US patients with AF; however, some limitations should be considered. As with all claims‐based analyses, errors in data collection may lead to misclassification of certain diagnoses (e.g., recurrent arrhythmias), events, or measures. Arrhythmias that were not associated with hospitalizations or ER visits were not captured in the current analysis. While those events impact patient experience, they would not contribute substantially toward HCRU burden, and it is not expected that a related underestimation of arrhythmia recurrence would differ meaningfully between the dronedarone and sotalol cohorts. While AADs come with standardized prescribing information, prescribing patterns may have varied based on local standard of care. This could lead to selection bias (e.g., therapies may be differently prescribed depending upon patient and disease characteristics such as severity of disease or off‐label treatment). Additionally, only prescriptions filled can be measured, but not actual consumption. However, it seems reasonable to assume that drug consumption is likely to closely reflect prescriptions rates, and there is no reason to believe that the magnitude of the association would differ appreciably between the cohorts. Furthermore, patient selection and outcome assessment was the same for the treatment cohorts and thus any differences are more likely due to drug prescription than other factors such as drug consumption.

Our findings may not generalize to US individuals without insurance, patients older or much younger than our data set (mean 61 years), and patients with AF in countries outside of the United States.

The analysis could be impacted by survival bias because post‐CA patients who died before a dronedarone or sotalol prescription claim were excluded. To assess any potential impact of survival bias, time from CA to first post‐CA AAD therapy initiation was assessed categorically (≤ 90 vs. > 90 days post‐CA) and continuously, and time from CA to first post‐CA AAD therapy initiation (continuous) was included as a matching criterion in the PSM. To avoid a skew on average HCRU, start of follow‐up was the day following CA, rather than the date of CA, to avoid capturing HCRU associated with the actual CA.

Inpatient prescription data were not available in the MarketScan databases, which is particularly relevant for the sotalol cohort, as sotalol is often initiated and dose‐adjusted in the inpatient setting [45]. Inpatient sotalol administration introduces survival bias in favor of sotalol because patients who discontinued sotalol in hospital owing to side effects would not be included. In addition, patients who discontinued sotalol but then initiated dronedarone as an outpatient would be subsequently captured (incorrectly) within the dronedarone cohort; if a patient initiated another AAD as an outpatient, they would not be eligible for the study. Index CA setting (inpatient/outpatient) remained unbalanced after PSM (ASD > 0.1), which could be related to patients initially receiving sotalol as an inpatient. However, there was a coding transition from CA being considered an inpatient procedure to largely an outpatient procedure around 2016, which fell during the study period. It is possible that when CA was mostly coded as “inpatient” pre‐2016, sotalol administered in‐hospital could have contributed to this imbalance for patients who continued on‐therapy. In‐hospital administration may also have led to more hospitalizations being captured in the sotalol cohort if patients remained on‐treatment and required dose adjustment during follow‐up.

Using ICD‐9/10 codes allowed specific patients to be selected who had undergone CA with a diagnosis for paroxysmal or persistent AF; however, we did not analyze subgroups of AF. Furthermore, technical aspects of the ablation procedure (e.g., energy source, lesion set) were not available and may impact outcomes. Claims data also lack some other measures relevant to analysis of AADs including measures of QTc and heart rate, for example. However, as dronedarone and sotalol both have class II and III properties and both are contraindicated among patients with bradycardia and prolonged QT, there is a low likelihood this would affect treatment decisions in either cohort. Furthermore, sotalol‐ and dronedarone‐treated cohorts were compared on the basis of expected similarities in eligible patients and drug classification, but our findings would not generalize to groups treated with other AADs post‐CA. Additional research would be needed to determine the impact of medication tolerability or adherence, health care costs, and copay on our observations, as these factors were outside the scope of this study.

## 5 Conclusions

In a large claims‐based cohort of patients with AF in the US, prescription of dronedarone or sotalol following CA occurred in about one‐quarter of patients. Matched patients with AF prescribed dronedarone post‐CA had routinely lower HCRU than those prescribed sotalol, principally a reduction in all‐cause and CV‐related hospitalizations. This pattern seen in the cohort overall was similar in patients new to dronedarone or sotalol therapy after CA, and in female and male patients, although some differences by sex were noted. Females treated with dronedarone post‐CA had fewer ER visits than those treated with sotalol, whereas no difference in ER visits was seen among males. Post‐CA management of AF remains a challenge. These findings can help guide clinical decision‐making when post‐CA antiarrhythmic treatment is indicated.

## Author Contributions

All authors contributed to study concept/design or contributed to data analysis/interpretation. All authors were involved in drafting the article and critical revision of the article. All authors approved the final version of the manuscript and take responsibility for the final content.

## Ethics Statement

All data in Merative MarketScan datasets are Health Insurance Portability and Accountability Act (HIPPA) (1996) compliant and deidentified to adhere with all relevant US regulations and privacy laws.

## Conflicts of Interest

Emily P. Zeitler: Travel and speaking support, Medtronic and Abbott; consulting support, Biosense Webster, Medtronic; nonfinancial research support, Sanofi, Biosense Webster; received support from the National Institute Of General Medical Sciences of the National Institutes of Health under Award Number P20GM148278. This work reflects the views of the authors and not any official views of the NIH/NIGMS. Dara Stein and Nicole Stamas: Employees of Evidera at the time of the study. Evidera received funding for this study from Sanofi. Ron Preblick, Shaum M. Kabadi, and David S. McKindley: Employees of Sanofi and may hold shares and/or stock options in the company. Jason Rashkin: Employee of Sanofi at the time of the study and may hold shares and/or stock options in the company. Samuel Huse: Current employee of Evidera. Evidera received funding for this study from Sanofi. Michael H. Kim: Consulting support from Sanofi.

## Supporting information

Supporting information.

## Data Availability

The data are not publicly available due to privacy or ethical restrictions. Qualified researchers may request access to data. Further details on Sanofi's data sharing criteria, eligible studies, and process for requesting access can be found at: https://www.vivli.org/.

## References

[1] L. Wang , F. Ze , J. Li , et al., “Trends of Global Burden of Atrial Fibrillation/Flutter From Global Burden of Disease Study 2017,” Heart 107 (2021): 881–887. 33148545 10.1136/heartjnl-2020-317656

[2] G. A. Roth , G. A. Mensah , C. O. Johnson , et al., “Global Burden of Cardiovascular Diseases and Risk Factors, 1990‐2019,” Journal of the American College of Cardiology 76 (2020): 2982–3021. 33309175 10.1016/j.jacc.2020.11.010 PMC7755038

[3] C. W. Tsao , A. W. Aday , Z. I. Almarzooq , et al., “Heart Disease and Stroke Statistics‐2022 Update,” Circulation 145 (2022): e153–e639. 35078371 10.1161/CIR.0000000000001052

[4] H. Li , X. Song , Y. Liang , et al., “Global, Regional, and National Burden of Disease Study of Atrial Fibrillation/Flutter, 1990–2019: Results From a Global Burden of Disease Study, 2019,” BMC Public Health 22 (2022): 2015. 36329400 10.1186/s12889-022-14403-2 PMC9632152

[5] R. B. Schnabel , X. Yin , P. Gona , et al., “50 Year Trends in Atrial Fibrillation Prevalence, Incidence, Risk Factors, and Mortality in the Framingham Heart Study: A Cohort Study,” Lancet 386 (2015): 154–162. 25960110 10.1016/S0140-6736(14)61774-8 PMC4553037

[6] S. Colilla , A. Crow , W. Petkun , D. E. Singer , T. Simon , and X. Liu , “Estimates of Current and Future Incidence and Prevalence of Atrial Fibrillation in the U.S. Adult Population,” American Journal of Cardiology 112 (2013): 1142–1147. 23831166 10.1016/j.amjcard.2013.05.063

[7] S. S. Chugh , R. Havmoeller , K. Narayanan , et al., “Worldwide Epidemiology of Atrial Fibrillation: A Global Burden of Disease 2010 Study,” Circulation 129 (2014): 837–847. 24345399 10.1161/CIRCULATIONAHA.113.005119 PMC4151302

[8] J. Zhang , S. P. Johnsen , Y. Guo , and G. Y. H. Lip , “Epidemiology of Atrial Fibrillation:Geographic/Ecological Risk Factors, Age, Sex, Genetics,” Cardiac Electrophysiology Clinics 13 (2021): 1–23. 33516388 10.1016/j.ccep.2020.10.010

[9] A. Deshmukh , M. Iglesias , R. Khanna , and T. Beaulieu , “Healthcare Utilization and Costs Associated With a Diagnosis of Incident Atrial Fibrillation,” Heart Rhythm O2 3 (2022): 577–586. 36340482 10.1016/j.hroo.2022.07.010 PMC9626881

[10] E. P. Zeitler , C. J. Ronk , A. Cockerham , S. Huse , D. S. McKindley , and M. H. Kim , “Healthcare Resource Utilization in Patients With Newly Diagnosed Atrial Fibrillation in the United States,” Expert Review of Pharmacoeconomics & Outcomes Research 22 (2022): 763–771. 35209794 10.1080/14737167.2022.2045955

[11] M. H. Kim , S. S. Johnston , B. C. Chu , M. R. Dalal , and K. L. Schulman , “Estimation of Total Incremental Health Care Costs in Patients With Atrial Fibrillation in the United States,” Circulation: Cardiovascular Quality and Outcomes 4 (2011): 313–320. 21540439 10.1161/CIRCOUTCOMES.110.958165

[12] L. Zhang , R. Gallagher , and L. Neubeck , “Health‐Related Quality of Life in Atrial Fibrillation Patients Over 65 Years: A Review,” European Journal of Preventive Cardiology 22 (2015): 987–1002. 24924742 10.1177/2047487314538855

[13] J. A. Joglar , M. K. Chung , A. L. Armbruster , et al., “2023 ACC/AHA/ACCP/HRS Guideline for the Diagnosis and Management of Atrial Fibrillation: A Report of the American College of Cardiology/American Heart Association Joint Committee on Clinical Practice Guidelines,” *Circulation* 149, no. 1 (2024): e1–e156. 10.1161/CIR.0000000000001193 PMC11095842 38033089

[14] G. Hindricks , T. Potpara , N. Dagres , et al., “2020 ESC Guidelines for the Diagnosis and Management of Atrial Fibrillation,” European Heart Journal 42 (2020): 373–498. 10.1093/eurheartj/ehaa612 32860505

[15] A. J. Camm , G. V. Naccarelli , S. Mittal , et al., “The Increasing Role of Rhythm Control in Patients With Atrial Fibrillation: JACC State‐of‐the‐Art Review,” Journal of the American College of Cardiology 79 (2022): 1932–1948. 35550691 10.1016/j.jacc.2022.03.337

[16] M. Schmidt , U. Dorwarth , D. Andresen , et al., “German Ablation Registry: Cryoballoon vs. Radiofrequency Ablation in Paroxysmal Atrial Fibrillation‐One‐Year Outcome Data,” Heart Rhythm 13 (2016): 836–844. 26681608 10.1016/j.hrthm.2015.12.007

[17] H. Van Brabandt , M. Neyt , and C. Devos , “Effectiveness of Catheter Ablation of Atrial Fibrillation in Belgian Practice: A Cohort Analysis on Administrative Data,” EP Europace 15 (2013): 663–668. 23388182 10.1093/europace/eut004

[18] E. Arbelo , J. Brugada , C. Blomström‐Lundqvist , et al., “Contemporary Management of Patients Undergoing Atrial Fibrillation Ablation: In‐Hospital and 1‐year Follow‐Up Findings From the ESC‐EHRA Atrial Fibrillation Ablation Long‐Term Registry,” European Heart Journal 38 (2017): 1303–1316. 28104790 10.1093/eurheartj/ehw564

[19] J. Roux , E. Zado , D. J. Callans , et al., “Antiarrhythmics After Ablation of Atrial Fibrillation (5A Study),” Circulation 120 (2009): 1036–1040. 19738139 10.1161/CIRCULATIONAHA.108.839639

[20] C. L. Malladi , D. Darden , O. Aldaas , et al., “Association Between Specific Antiarrhythmic Drug Prescription in the Post‐Procedural Blanking Period and Recurrent Atrial Arrhythmias After Catheter Ablation for Atrial Fibrillation,” PLoS One 16 (2021): e0253266. 34166392 10.1371/journal.pone.0253266 PMC8224843

[21] “Cordarone (Amiodarone HCl) Tablets ,” 2010, https://www.accessdata.fda.gov/drugsatfda_docs/label/2010/018972s042lbl.pdf.

[22] C. T. January , L. S. Wann , J. S. Alpert , et al., “2014 AHA/ACC/HRS Guideline for the Management of Patients With Atrial Fibrillation: A Report of the American College of Cardiology/American Heart Association Task Force on Practice Guidelines and the Heart Rhythm Society,” Circulation 130 (2014): e199–e267. 24682347 10.1161/CIR.0000000000000041 PMC4676081

[23] J. Y. Le Heuzey , G. M. De Ferrari , D. Radzik , M. Santini , J. Zhu , and J. M. Davy , “A Short‐Term, Randomized, Double‐Blind, Parallel‐Group Study to Evaluate the Efficacy and Safety of Dronedarone Versus Amiodarone in Patients With Persistent Atrial Fibrillation: The DIONYSOS Study,” Journal of Cardiovascular Electrophysiology 21 (2010): 597–605. 20384650 10.1111/j.1540-8167.2010.01764.x

[24] J. P. Singh , C. Blomström‐Lundqvist , M. P. Turakhia , et al., “Dronedarone Versus Sotalol in Patients With Atrial Fibrillation: A Systematic Literature Review and Network Meta‐Analysis,” Clinical Cardiology 46 (2023): 589–597. 37025083 10.1002/clc.24011 PMC10270269

[25] M. Gulizia , S. Mangiameli , S. Orazi , et al., “A Randomized Comparison of Amiodarone and Class IC Antiarrhythmic Drugs to Treat Atrial Fibrillation in Patients Paced for Sinus Node Disease: The Prevention Investigation and Treatment: A Group for Observation and Research on Atrial Arrhythmias (PITAGORA) Trial,” American Heart Journal 155 (2008): 100–107. 18082498 10.1016/j.ahj.2007.08.033

[26] M. Vamos , H. Calkins , P. R. Kowey , et al., “Efficacy and Safety of Dronedarone in Patients With a Prior Ablation for Atrial Fibrillation/Flutter: Insights From the ATHENA Study,” Clinical Cardiology 43 (2020): 291–297. 31872901 10.1002/clc.23309 PMC7068068

[27] J. M. Wharton , J. P. Piccini , A. Koren , S. Huse , and C. J. Ronk , “Comparative Safety and Effectiveness of Sotalol Versus Dronedarone After Catheter Ablation for Atrial Fibrillation,” Journal of the American Heart Association 11 (2022): e020506. 35060388 10.1161/JAHA.120.020506 PMC9238499

[28] “Merative^TM^ MarketScan® Research Databases,” 2022, https://www.merative.com/content/dam/merative/documents/brief/Marketscan_explainer_general.pdf.

[29] M. Sturkenboom and T. Schink , ed., Databases for Pharmacoepidemiological Research (Switzerland AG: Springer Nature, 2021).

[30] H. Quan , V. Sundararajan , P. Halfon , et al., “Coding Algorithms for Defining Comorbidities in ICD‐9‐CM and ICD‐10 Administrative Data,” Medical Care 43 (2005): 1130–1139. 16224307 10.1097/01.mlr.0000182534.19832.83

[31] G. Y. H. Lip , R. Nieuwlaat , R. Pisters , D. A. Lane , and H. J. G. M. Crijns , “Refining Clinical Risk Stratification for Predicting Stroke and Thromboembolism in Atrial Fibrillation Using a Novel Risk Factor‐Based Approach,” Chest 137 (2010): 263–272. 19762550 10.1378/chest.09-1584

[32] D. L. Steen , I. Khan , L. Becker , et al., “Patterns and Predictors of Lipid‐Lowering Therapy in Patients With Atherosclerotic Cardiovascular Disease And/Or Diabetes Mellitus in 2014: Insights From a Large US Managed‐Care Population,” Clinical Cardiology 40 (2017): 155–162. 28026031 10.1002/clc.22641 PMC6490402

[33] M. H. Kim , D. Klingman , J. Lin , and D. S. Battleman , “Patterns and Predictors of Discontinuation of Rhythm‐Control Drug Therapy in Patients With Newly Diagnosed Atrial Fibrillation,” Pharmacotherapy: The Journal of Human Pharmacology and Drug Therapy 29 (2009): 1417–1426. 10.1592/phco.29.12.1417 19947801

[34] F. Guerra , S. H. Hohnloser , P. R. Kowey , et al., “Efficacy and Safety of Dronedarone in Patients Previously Treated With Other Antiarrhythmic Agents,” Clinical Cardiology 37 (2014): 717–724. 25470298 10.1002/clc.22342 PMC6647669

[35] A. B. Curtis , E. P. Zeitler , A. Malik , et al., “Efficacy and Safety of Dronedarone Across Age and Sex Subgroups: A Post Hoc Analysis of the ATHENA Study Among Patients With Non‐Permanent Atrial Fibrillation/Flutter,” Europace: European Pacing, Arrhythmias, and Cardiac Electrophysiology 24 (2022): 1754–1762. 10.1093/europace/euab208 PMC9681127 34374766

[36] S. H. Hohnloser , H. J. G. M. Crijns , M. van Eickels , et al., “Effect of Dronedarone on Cardiovascular Events in Atrial Fibrillation,” New England Journal of Medicine 360 (2009): 668–678. 19213680 10.1056/NEJMoa0803778

[37] E. P. Zeitler , J. E. Poole , C. M. Albert , et al., “Arrhythmias in Female Patients: Incidence, Presentation and Management,” Circulation Research 130 (2022): 474–495. 35175839 10.1161/CIRCRESAHA.121.319893

[38] R. B. Schnabel , L. Pecen , F. M. Ojeda , et al., “Gender Differences in Clinical Presentation and 1‐year Outcomes in Atrial Fibrillation,” Heart 103 (2017): 1024–1030. 28228467 10.1136/heartjnl-2016-310406 PMC5529986

[39] A. Israeli , D. Gal , A. Younis , et al., “Sex‐Differences in Atrial Fibrillation Patients: Bias or Proper Management?,” Vascular Health and Risk Management 18 (2022): 347–358. 35546968 10.2147/VHRM.S366285 PMC9084509

[40] M. A. Hernán and J. M. Robins , “Using Big Data to Emulate a Target Trial When a Randomized Trial Is Not Available,” American Journal of Epidemiology 183 (2016): 758–764. 26994063 10.1093/aje/kwv254 PMC4832051

[41] K. Yoshida , D. H. Solomon , and S. C. Kim , “Active‐Comparator Design and New‐User Design in Observational Studies,” Nature Reviews Rheumatology 11 (2015): 437–441. 25800216 10.1038/nrrheum.2015.30 PMC4486631

[42] V. Allan , S. V. Ramagopalan , J. Mardekian , et al., “Propensity Score Matching and Inverse Probability of Treatment Weighting to Address Confounding by Indication in Comparative Effectiveness Research of Oral Anticoagulants,” Journal of Comparative Effectiveness Research 9 (2020): 603–614. 32186922 10.2217/cer-2020-0013

[43] P. C. Austin , “An Introduction to Propensity Score Methods for Reducing the Effects of Confounding in Observational Studies,” Multivariate Behavioral Research 46 (2011): 399–424. 21818162 10.1080/00273171.2011.568786 PMC3144483

[44] P. R. Rosenbaum and D. B. Rubin , “The Central Role of the Propensity Score in Observational Studies for Causal Effects,” Biometrika 70 (1983): 41–55.

[45] M. K. Chung , R. A. Schweikert , B. L. Wilkoff , et al., “Is Hospital Admission for Initiation of Antiarrhythmic Therapy With Sotalol for Atrial Arrhythmias Required?,” Journal of the American College of Cardiology 32 (1998): 169–176. 9669266 10.1016/s0735-1097(98)00189-2

