# Supplementary material for: Health Care Resource Utilization With Dronedarone Versus Sotalol Following Catheter Ablation in Adults With Atrial Fibrillation
Source: Clin Cardiol. 2025 Jan 15;48(1):e70064. doi: 10.1002/clc.70064 (PMC11733741; doi:10.1002/clc.70064)
Supplement: Supplementary file 1 — Supporting information. [file CLC-48-e70064-s001.docx]

Health care resource utilization with dronedarone versus sotalol following catheter ablation in adults with atrial fibrillation

Emily P. Zeitler, MD, MHS^1^ | Dara Stein, MSc^2^* | Ron Preblick, PharmD, MPH^3^ | Shaum M. Kabadi, PhD, MPH^3^ | David S. McKindley, PharmD^3^ | Jason Rashkin, MD ^3^ | Samuel Huse, PhD^4^ | Nicole Stamas, MSc^5^ | Michael H. Kim, MD, MMM^6^

^1^Dartmouth-Hitchcock Medical Center, Lebanon, NH, USA

^2^Evidera, London, UK (*at time of study)

^3^Sanofi, Bridgewater, NJ, USA

^4^Evidera, Cambridge, MA, USA

^5^Evidera, Seattle, WA, USA

^6^Creighton University School of Medicine and CHI Health, Omaha, NE, USA

**Corresponding author**: Emily P. Zeitler, MD, MHS

email: [Emily.P.Zeitler@hitchcock.org](mailto:Emily.P.Zeitler@hitchcock.org)

7 Tables: 4 Figures

### Supplementary Table S1. Definition of variables used for PSM.

| **Variable** | **Classification/Definition** |
| --- | --- |
| Age at index (years; rounded to integer) | Continuous |
| Sex | Male, Female (Categorical) |
| Region | Northeast, North Central, South, West (Categorical) |
| Enrollment in Medicare Supplemental | Yes/No (Binary) |
| **Comorbidities** | |
| Coronary heart disease | Yes/No (Binary) |
| COPD | Yes/No (Binary) |
| Diabetes | Yes/No (Binary) |
| Heart failure | Yes/No (Binary) |
| Hypertension | Yes/No (Binary) |
| Ischemic stroke | Yes/No (Binary) |
| Myocardial infarction | Yes/No (Binary) |
| Peripheral artery disease | Yes/No (Binary) |
| Venous thromboembolism | Yes/No (Binary) |
| Vascular disease | Yes/No (Binary) |
| Time from CA to first post-CA AAD therapy | Continuous |
| History of atrial flutter at any time prior to index CA | Yes/No (Binary) |
| CCI | Continuous |
| CHA_2_DS_2_-VASc Score | Continuous |
| Baseline procedures | Binary (yes/no): At least one |
| Electrocardiogram | Yes/No (Binary) |
| Cardioversion | Yes/No (Binary) |
| Baseline medication use | Yes/No (Binary) |
| Any prior AAD use during baseline period | Yes/No (Binary) |
| DOACs | Yes/No (Binary) |
| VKAs/warfarin | Yes/No (Binary) |
| Antiplatelet therapy (P2Y12 inhibitors) | Yes/No (Binary) |
| Rate control medication (beta blockers, CCB, NDHP, DHP, digoxin) | Yes/No (Binary) |
| Antihypertensives (ACE inhibitors, ARBs, Aldosterone, loop diuretics) | Yes/No (Binary) |
| **Baseline HCRU** | |
| Mean number of all-cause outpatient visits | Continuous |
| Mean number of ATA-/AF-related outpatient visits | Continuous |
| Mean number of CV-related outpatient visits | Continuous |
| At least one inpatient hospitalization | Yes/No (Binary) |
| At least one ER visit | Yes/No (Binary) |
| AAD, antiarrhythmic drug; ACE, angiotensin converting enzyme; ARB, angiotensin receptor blocker; ATA/AF, atrial tachyarrhythmia/atrial fibrillation; CCB, calcium channel blocker; CCI, Charlson Comorbidity Index; CHA_2_DS_2_-VASc, congestive heart failure, hypertension, age ≥75 years [doubled], diabetes, stroke/transient ischemic attack/thromboembolism [doubled], vascular disease [prior myocardial infarction, peripheral artery disease, or aortic plaque], age 65–74 years, sex category [female]); COPD, chronic obstructive pulmonary disease; CV, cardiovascular; DOAC, direct-acting oral anticoagulant; DHP, dihydropyridine; ER, emergency room; HCRU, health care resource utilization; NDHP, non-dihydropyridine; PSM, propensity score matching; VKA, vitamin K antagonist. | |

.

### Supplementary Table S2. Definition of HCRU used in study of Merative^TM^ MarketScan^®^ Commercial and Medicare Supplemental databases.

| **Primary Measures** | **Definition** |
| --- | --- |
| **All-cause HCRU** | |
| Hospitalization | Hospital stay for any cause |
| ER visit | ER visit for any cause |
| Outpatient office visit | Outpatient office visit as defined below for any cause |
| Other outpatient services | Other outpatient services as defined below for any cause |
| **Secondary Measures** | **Definition** |
| **CV-related HCRU** | |
| Hospitalization | Hospitalizations with a primary ICD-9/10 diagnosis code related to AF, atrial tachyarrhythmias, ischemic stroke/TIA, myocardial infarction, ventricular arrhythmias, bradycardia, or HF |
| ER visit | ER visits with a primary ICD-9/10 diagnosis code related to AF, atrial tachyarrhythmias, ischemic stroke/TIA, myocardial infarction, ventricular arrhythmias, bradycardia, or HF |
| Outpatient office visit | Outpatient visits with a primary ICD-9/10 diagnosis code related to AF, atrial tachyarrhythmias, ischemic stroke/TIA, myocardial infarction, ventricular arrhythmias, bradycardia, or HF |
| Other outpatient services | Other outpatient services with a primary ICD-9/10 diagnosis code related to AF, atrial tachyarrhythmias, ischemic stroke/TIA, myocardial infarction, ventricular arrhythmias, bradycardia, or HF |
| Prescription | List of CV-related drugs: AADs (dronedarone, sotalol, amiodarone, flecainide, propafenone, dofetilide), Beta blockers, Calcium channel blockers (NDHP, DHP), Digoxin, DOACs, VKA/warfarin, ASAs, P2Y12 inhibitors (antiplatelets), ACE inhibitors, ARBs, Aldosterone, Loop diuretics |
| Hospitalization for ACS/MI | Hospitalization containing a primary ICD-9/10 diagnosis code for either MI or unstable angina |
| Hospitalization for HF | Hospitalization containing a primary ICD-9/10 diagnosis code for HF |
| Hospitalization for ischemic stroke | Hospitalization containing a primary ICD-9/10 diagnosis code for IS |
| **CV-related procedures** | |
| Cardio-defibrillator implantation | Claims containing a CPT or ICD-9/ICD-10-PCS procedure code for insertion of a cardio-defibrillator |
| Pacemaker implantation | Claims containing a CPT or ICD-9/ICD-10-PCS procedure code for implantation (or insertion) of a pacemaker |
| **ATA/AF-related HCRU** | |
| Hospital | Hospitalizations with a primary ICD-9/10 diagnosis code related to AF/atrial tachyarrhythmias |
| ER visits | ER visits with a primary ICD-9/10 diagnosis code related to AF/atrial tachyarrhythmias |
| Outpatient | Outpatient visits with a primary ICD-9/10 diagnosis code related to AF/atrial tachyarrhythmias |
| Other outpatient services | Other outpatient services as defined per Table A4 in the Appendix with a primary ICD-9/10 diagnosis code related to AF/atrial tachyarrhythmias |
| Prescription | Prescriptions for AAD or anticoagulants will be considered “AF-related”. List of AF-related drugs: AADs (dronedarone, sotalol, amiodarone, flecainide, propafenone, dofetilide), Beta blockers, Calcium channel blockers (NDHP, DHP), Digoxin, DOACs, VKA/warfarin, P2Y12 inhibitors (antiplatelets) |
| **AF-related procedures** |  |
| Cardioversion procedures | Claims containing a CPT or ICD-9/ICD-10-PCS procedure code for cardioversion procedure |
| Repeat catheter ablation procedures | Claims containing a CPT or ICD-9/ICD-10-PCS procedure code for catheter ablation |
| **Outpatient HCRU** | |

| **Place of service** | **Classification** |
| --- | --- |
| Office | Outpatient Office Visit |
| Mobile Unit | Outpatient Office Visit |
| Walk-in Retail Health Clinic | Outpatient Office Visit |
| Outpatient Hospital-Off Campus | Outpatient Office Visit |
| Independent Clinic | Outpatient Office Visit |
| Federally Qualified Health Ctr | Outpatient Office Visit |
| State/Local Public Health Clin | Outpatient Office Visit |
| Rural Health Clinic | Outpatient Office Visit |
| Outpatient (NEC) | Outpatient Office Visit |
| Patient Home | Other Outpatient Service |
| Other Unlisted Facility | Other Outpatient Service |
| Pharmacy | Other Outpatient Service |
| School | Other Outpatient Service |
| Assisted Living Facility | Other Outpatient Service |
| Place of Employment-Worksite | Other Outpatient Service |
| Urgent Care Facility | Other Outpatient Service |
| Inpatient Hospital | Other Outpatient Service |
| Outpatient Hospital-On Campus | Other Outpatient Service |
| Ambulatory Surgical Center | Other Outpatient Service |
| Birthing Center | Other Outpatient Service |
| Skilled Nursing Facility | Other Outpatient Service |
| Nursing Facility | Other Outpatient Service |
| Custodial Care Facility | Other Outpatient Service |
| Hospice | Other Outpatient Service |
| Ambulance (land) | Other Outpatient Service |
| Ambulance (air or water) | Other Outpatient Service |
| Inpatient Psychiatric Facility | Other Outpatient Service |
| Psychiatric Facility Partial Hospital | Other Outpatient Service |
| Community Mental Health Center | Other Outpatient Service |
| Residential Substance Abuse Facility | Other Outpatient Service |
| Psychiatric Residential Treatment Center | Other Outpatient Service |
| Non-resident Substance Abuse Facility | Other Outpatient Service |
| Mass Immunization Center | Other Outpatient Service |
| Comprehensive Inpatient Rehabilitation Facility | Other Outpatient Service |
| Comprehensive Outpatient Rehabilitation Facility | Other Outpatient Service |
| End-Stage Renal Disease Facility | Other Outpatient Service |
| Independent Laboratory | Other Outpatient Service |
| Pharmacy | Other Outpatient Service |

*AF ICD-9 code: 427.31; ICD-10 codes: I48.0, I48.1, I48.2, I48.91, I48.11, I48.19, I48.20, I48.21.

AAD, antiarrhythmic drugs; ACS, acute coronary syndrome; AF, atrial fibrillation; CPT, Current Procedural Terminology; CV, cardiovascular; CVD, cardiovascular disease; ER, emergency room; HCRU, health care resource utilization; HF, heart failure; ICD-9/10, International Classification of Diseases, Ninth/10^th^ Revision; LOS, length of stay; NA, not applicable; PCS, procedure coding system; TIA, transient ischemic attack.

### Supplemental Table S3. Characteristics of (A) female and (B) male subgroups of dronedarone and sotalol cohorts, after PSM.

| 1. **Female** | | | | 1. **Male** | | |
| --- | --- | --- | --- | --- | --- | --- |
| **Characteristics**  **Data are mean±SD or *n* (%)** | **After PSM** | | **ASD^a^** | **After PSM** | | **ASD^a^** |
|  | **Dronedarone**  **(*n*= 460)** | **Sotalol**  **(*n*= 460)** |  | **Dronedarone**  **(*n*= 1115)** | **Sotalol**  **(*n*= 1115)** |  |
| Age at index^b^, y | 63.8±9.7 | 63.9±8.9 | 0.015 | 60.3±9.0 | 60.4±8.7 | 0.008 |
| **Region**^b^ | | | | | | |
| Northeast | 113(24.6) | 106(23.0) | 0.036 | 267(23.9) | 252(22.6) | 0.032 |
| North Central | 80(17.4) | 82(17.8) | 0.011 | 225(20.2) | 237(21.3) | 0.027 |
| South | 202(43.9) | 198(43.0) | 0.018 | 446(40.0) | 444(39.8) | 0.004 |
| West | 64(13.9) | 73(15.9) | 0.055 | 169(15.2) | 173(15.5) | 0.010 |
| Unknown | 1(0.2) | 1(0.2) | 0.000 | 8(0.7) | 9(0.8) | 0.010 |
| Urban residency | 371(80.7) | 342(74.3) | 0.151 | 903(81.0) | 868(77.8) | 0.078 |
| Medicare supplemental^b^ | 274(59.6) | 275(59.8) | 0.004 | 834(74.8) | 838(75.2) | 0.008 |
| **Selected comorbidities^c.^** | | | | | | |
| Hypertension^b^ | 338(73.5) | 350(76.1) | 0.060 | 776(69.6) | 779(69.9) | 0.006 |
| CHD^b^ | 162(35.2) | 170(37.0) | 0.036 | 449(40.3) | 446(40.0) | 0.006 |
| COPD^b^ | 91(19.8) | 85(18.5) | 0.033 | 61(5.5) | 70(6.3) | 0.034 |
| Diabetes^b^ | 91(19.8) | 93(20.2) | 0.011 | 201(18.0) | 203(18.2) | 0.005 |
| Heart failure^b^ | 67(14.6) | 67(14.6) | 0.000 | 129(11.6) | 145(13.0) | 0.044 |
| PAD^b^ | 32(7.0) | 38(8.3) | 0.049 | 91(8.2) | 88(7.9) | 0.010 |
| Ischemic stroke^b^ | 15(3.3) | 17(3.7) | 0.024 | 34(3.0) | 36(3.2) | 0.010 |
| Myocardial infarction^b^ | 9(2.0) | 7(1.5) | 0.033 | 39(3.5) | 44(3.9) | 0.024 |
| Venous thromboembolism^b^ | 9(2.0) | 9(2.0) | 0.000 | 17(1.5) | 19(1.7) | 0.014 |
| Vascular disease^b^ | 7(1.5) | 10(2.2) | 0.048 | 23(2.1) | 23(2.1) | 0.000 |
| History of AFL^b,d^ | 144(31.3) | 153(33.3) | 0.042 | 390(35.0) | 412(37.0) | 0.041 |
| CCI^b,e^ | 0.8±1.2 | 0.8±1.3 | 0.000 | 0.7±1.3 | 0.8±1.2 | 0.017 |
| CHA_2_DS_2_-VASc score^b,e^ | 2.8±1.3 | 2.8±1.3 | 0.030 | 1.4±1.1 | 1.5±1.2 | 0.022 |
| **Baseline procedures^b^** | | | | | | |
| ECG (≥ 1) | 455(98.9) | 454(98.7) | 0.020 | 1,103(98.9) | 1,103(98.9) | 0.000 |
| Number of ECG | 5.6±3.7 | 6.1±4.1 | 0.126 | 5.6±3.7 | 6.0±3.7 | 0.091 |
| Cardioversion (≥ 1) | 140(30.4) | 160(34.8) | 0.093 | 423(37.9) | 431(38.7) | 0.015 |
| **CA setting** | | | | | | |
| Outpatient CA | 420(91.3) | 387(84.1) | 0.220 | 1036(92.9) | 960(86.1) | 0.224 |
| Inpatient CA | 40(8.7) | 73(15.9) | 0.220 | 79(7.1) | 155(13.9) | 0.224 |
| **Baseline medication use**^b^ | | | | | | |
| Any AAD prior during baseline | 373(81.1) | 379(82.4) | 0.034 | 861(77.2) | 875(78.5) | 0.030 |
| Any DOAC | 328(71.3) | 330(71.7) | 0.010 | 814(73.0) | 807(72.4) | 0.014 |
| Beta blockers | 336(73.0) | 330(71.7) | 0.029 | 763(68.4) | 751(67.4) | 0.023 |
| CCB (DHP or NDHP) | 199(43.3) | 211(45.9) | 0.053 | 420(37.7) | 420(37.7) | 0.000 |
| ARBs | 106(23.0) | 111(24.1) | 0.026 | 200(17.9) | 194(17.4) | 0.014 |
| VKA/warfarin | 105(22.8) | 103(22.4) | 0.010 | 232(20.8) | 236(21.2) | 0.009 |
| ACE inhibitors | 90(19.6) | 92(20.0) | 0.011 | 324(29.1) | 310(27.8) | 0.028 |
| Loop diuretics | 79(17.2) | 77(16.7) | 0.012 | 125(11.2) | 119(10.7) | 0.017 |
| Digoxin | 59(12.8) | 58(12.6) | 0.007 | 108(9.7) | 112(10.0) | 0.012 |
| P2Y12 inhibitors | 35(7.6) | 36(7.8) | 0.008 | 129(11.6) | 124(11.1) | 0.014 |
| Aldosterone | 21(4.6) | 18(3.9) | 0.032 | 26(2.3) | 33(3.0) | 0.039 |
| Time from CA to first AAD Rx^b^ | 64.4±181.9 | 64.0±181.6 | 0.002 | 51.9±166.4 | 54.6±157.5 | 0.017 |
| **Baseline HCRU** | | | | | | |
| Outpatient visits^b^ | 22.1±15.4 | 22.5±16.3 | 0.025 | 18.5±14.7 | 18.5±14.8 | 0.002 |
| ATA/AF-related outpatient visits^b^ | 5.4±3.8 | 5.4±4.1 | 0.021 | 5.2±3.9 | 5.2±3.9 | 0.006 |
| CV-related outpatient visits^b^ | 5.5±4.0 | 5.6±4.2 | 0.023 | 5.3±4.0 | 5.3±3.9 | 0.010 |
| ≥ 1 ATA/AF-related hospitalization^b,f^ | - | - | - | 187±16.8 | 219±19.6 | 0.074 |
| ≥ 1 inpatient hospitalization^b^ | 167(36.3) | 170(37.0) | 0.014 | 296(26.5) | 332(29.8) | 0.072 |
| ≥ 1 ER visit^b^ | 206(44.8) | 205(44.6) | 0.004 | 359(32.2) | 362(32.5) | 0.006 |
| ^a^A covariate was considered balanced after PSM if ASD between treatment cohorts was ≤0.1 (10%). ^b^Included in the propensity score model (detailed in **Table S1**). ^c^Selected comorbidities recorded in 1-year pre-index period. ^d^At any time prior to index CA. ^e^CHA_2_DS_2_-VASc and CCI scores calculated during baseline period were continuous. ^f^Males were additionally matched on ≥1 ATA/AF-related hospitalization at baseline, females were not.  AAD, antiarrhythmic drug; ACE, angiotensin converting enzyme; AFL, atrial flutter; ARB, angiotensin receptor blocker; ASD, absolute standardized difference; ATA/AF, atrial tachyarrhythmia/atrial fibrillation; CA, catheter ablation; CCB, calcium channel blocker; CCI, Charlson Comorbidity Index; CHD, coronary heart disease; COPD, chronic obstructive pulmonary disease; CV, cardiovascular; DOAC; direct-acting oral anticoagulant; DHP, dihydropyridine; ECG, electrocardiogram; ER, emergency room; HCRU, health care resource utilization; NDHP, non-dihydropyridine; PAD, peripheral arterial disease; PSM, propensity score matching; Rx, prescription; SD, standard deviation; VKA, vitamin K. | | | | | | |

### Supplemental Table S4. Characteristics of patients with AF new to their index AAD (dronedarone or sotalol), among treatment cohorts after PSM.

| **Characteristics**  **Data mean±SD or *n* (%)** | **After PSM** | | **ASD^a^** |
| --- | --- | --- | --- |
|  | **Dronedarone**  **(*n*= 549)** | **Sotalol**  **(*n*= 549)** |  |
| Age at index,^b^ y | 61.7±9.5 | 61.4±9.3 | 0.035 |
| **Sex^b^** | | | |
| Females | 145 (26.4) | 149 (27.1) | 0.017 |
| Male | 404 (73.6) | 400 (72.9) | 0.017 |
| **Region**^b^ | | | |
| Northeast | 148 (27.0) | 139 (25.3) | 0.037 |
| North Central | 100 (18.2) | 104 (18.9) | 0.019 |
| South | 204 (37.2) | 202 (36.8) | 0.008 |
| West | 94 (17.1) | 100 (18.2) | 0.029 |
| Unknown | 3 (0.5) | 4 (0.7) | 0.023 |
| Urban residency | 463 (84.3) | 436 (79.4) | 0.128 |
| Medicare supplemental^b^ | 370 (67.4) | 383 (69.8) | 0.051 |
| **Selected comorbidities^c^** | | | |
| Hypertension^b^ | 393 (71.6) | 388 (70.7) | 0.020 |
| CHD^b^ | 217 (39.5) | 206 (37.5) | 0.041 |
| Diabetes^b^ | 106 (19.3) | 107 (19.5) | 0.005 |
| Heart failure^b^ | 75 (13.7) | 75 (13.7) | 0.000 |
| PAD^b^ | 41 (7.5) | 42 (7.7) | 0.007 |
| COPD^b^ | 26 (4.7) | 32 (5.8) | 0.049 |
| Ischemic stroke^b^ | 22 (4.0) | 18 (3.3) | 0.039 |
| Myocardial infarction^b^ | 20 (3.6) | 18 (3.3) | 0.020 |
| Vascular disease^b^ | 13 (2.4) | 13 (2.4) | 0.000 |
| Venous thromboembolism^b^ | 5 (0.9) | 7 (1.3) | 0.035 |
| History of AFL^b,d^ | 198 (36.1) | 191 (34.8) | 0.027 |
| CCI^b,e^ | 0.8±1.2 | 0.8±1.1 | 0.006 |
| CHA_2_DS_2_-VASc score^b,e^ | 1.9±1.4 | 1.8±1.3 | 0.027 |
| **Baseline procedures^b^** | | | |
| ECG (≥ 1) | 545 (99.3) | 542 (98.7) | 0.055 |
| Number of ECG | 5.5±3.9 | 5.4±3.3 | 0.047 |
| Cardioversion (≥ 1) | 207 (37.7) | 222 (40.4) | 0.056 |
| **CA setting** |  |  |  |
| Outpatient CA | 507 (92.3) | 415 (75.6) | 0.469 |
| Inpatient CA | 42 (7.7) | 134 (24.4) | 0.469 |
| **Baseline medication use^b^** | | | |
| Any AAD prior during baseline | 243 (44.3) | 248 (45.2) | 0.018 |
| Any DOAC | 395 (71.9) | 391 (71.2) | 0.016 |
| VKA/warfarin | 114 (20.8) | 122 (22.2) | 0.036 |
| P2Y12 inhibitors | 61 (11.1) | 59 (10.7) | 0.012 |
| Beta blockers | 417 (76.0) | 418 (76.1) | 0.004 |
| CCBs (DHP or NDHP) ^b^ | 218 (39.7) | 221 (40.3) | 0.011 |
| Digoxin | 70 (12.8) | 70 (12.8) | 0.000 |
| ACE inhibitors | 159 (29.0) | 165 (30.1) | 0.024 |
| ARBs | 111 (20.2) | 99 (18.0) | 0.056 |
| Aldosterone | 17 (3.1) | 17 (3.1) | 0.000 |
| Loop diuretics | 72 (13.1) | 76 (13.8) | 0.021 |
| Time from CA to first AAD Rx^b^ | 83.8±242.6 | 85.1±227.1 | 0.006 |
| **Baseline HCRU** | | | |
| Outpatient visits^b^ | 18.7±14.9 | 19.1±14.4 | 0.029 |
| ATA/AF-related outpatient visits^b^ | 5.0±3.9 | 5.2±4.3 | 0.042 |
| CV-related outpatient visits^b^ | 5.1±4.0 | 5.3±4.3 | 0.041 |
| ≥ 1 inpatient hospitalization^b^ | 165 (30.1) | 158 (28.8) | 0.028 |
| ≥ 1 ER visit ^b^ | 185 (33.7) | 183 (33.3) | 0.008 |
| ^a^A covariate was considered balanced after PSM if ASD between treatment cohorts was ≤0.1 (10%). ^b^Included in the propensity score model (detailed in **Table S1**). ^c^Selected comorbidities recorded in 1-year pre-index period. ^d^At any time prior to index CA.  ^e^CHA_2_DS_2_-VASc and CCI scores calculated during baseline period were continuous. ^f^Males were additionally matched on ATA/AF hospitalizations.  AAD, antiarrhythmic drug; ACE, angiotensin converting enzyme; AFL, atrial flutter; ARB, angiotensin receptor blocker; ASD, absolute standardized difference; ATA/AF, atrial tachyarrhythmia/atrial fibrillation; CA, catheter ablation; CCB, calcium channel blocker; CCI, Charlson Comorbidity Index; CHD, coronary heart disease; COPD, chronic obstructive pulmonary disease; CV, cardiovascular; DOAC; direct-acting oral anticoagulant; DHP, dihydropyridine; ECG, electrocardiogram; ER, emergency room; HCRU, health care resource utilization; NDHP, non-dihydropyridine; PAD, peripheral arterial disease; PSM, propensity score matching; Rx, prescription; SD, standard deviation; VKA, vitamin K antagonist. | | | |

### Supplemental Table S5. AAD use after discontinuation in the dronedarone and sotalol cohorts, after PSM.

| **Cohort** | **Next AAD after discontinuation** | **No. patients (n)** | **Mean time from discontinuation to new AAD (days)** | **Median time from discontinuation to new AAD (days)** | **Min time from discontinuation to new AAD (days)** | **Max time from discontinuation to new AAD (days)** |
| --- | --- | --- | --- | --- | --- | --- |
| dronedarone | No further AADs | 1026 | NA | NA | NA | NA |
| dronedarone | dronedarone | 214 | 184.2 | 77 | 31 | 2058 |
| dronedarone | amiodarone | 127 | 115.7 | 0 | 0 | 1669 |
| dronedarone | flecainide | 66 | 108.2 | 0 | 0 | 1054 |
| dronedarone | sotalol | 58 | 78.1 | 4 | 0 | 990 |
| dronedarone | propafenone | 34 | 108.2 | 0 | 0 | 1225 |
| dronedarone | dofetilide | 29 | 120.6 | 4 | 0 | 1538 |
| dronedarone | disopyramide | 1 | 0 | 0 | 0 | 0 |
|  |  |  |  |  |  |  |
| sotalol | No further AADs | 960 | NA | NA | NA | NA |
| sotalol | sotalol | 350 | 117.8 | 61 | 31 | 1335 |
| sotalol | amiodarone | 97 | 112.4 | 0 | 0 | 2192 |
| sotalol | flecainide | 63 | 115.1 | 0 | 0 | 894 |
| sotalol | dofetilide | 41 | 31.9 | 0 | 0 | 564 |
| sotalol | dronedarone | 22 | 144.7 | 51 | 0 | 740 |
| sotalol | propafenone | 15 | 68.7 | 0 | 0 | 685 |
| sotalol | disopyramide | 2 | 30.5 | 7 | 7 | 54 |
| sotalol | quinidine | 1 | 0 | 0 | 0 | 0 |

AAD, antiarrhythmic drugs; AF, atrial fibrillation; CA, catheter ablation; NA, not applicable.

.

### Supplemental Table S6. HCRU prevalence rates in female and male subgroups, among dronedarone and sotalol cohorts after PSM.

|  | **Dronedarone (*n*= 460)^a^** | | | **Sotalol (*n*= 460)^a^** | | |  |
| --- | --- | --- | --- | --- | --- | --- | --- |
| **Outcomes** | **Patients with event, *n* (%)** | **Total events** | **Rate per 100 PY (95% CI)** | **Patients with event, *n* (%)** | **Total events** | **Rate per 100 PY (95% CI)** | ***P* value** |
| **Female** |  |  |  |  |  |  |  |
| Hospitalizations | 177 (38.5) | 308 | 27.6 (24.5–30.7) | 190 (41.3) | 347 | 31.9 (28.5–35.2) | .065 |
| ER visits | 215 (46.7) | 641 | 57.4 (53.0–61.9) | 213 (46.3) | 819 | 75.3 (70.1–80.4) | <.001 |
| Outpatient office visits^b^ | 450 (97.8) | 24074 | 2156.6 (2129.4–2183.8) | 458 (99.6) | 24180 | 2221.6 (2193.6–2249.6) | .001 |
| Other outpatient services^c^ | 457 (99.3) | 13624 | 1220.5 (1200.0–1241.0) | 453 (98.5) | 14806 | 1360.4 (1338.4–1382.3) | <.001 |
| CV-related hospitalization | 92 (20.0) | 115 | 10.3 (8.4 –12.2) | 104 (22.6) | 149 | 13.7 (11.5 –15.9) | .022 |
| CV-related ER visits | 48 (10.4) | 115 | 10.3 (8.4 –12.2) | 70 (15.2) | 153 | 14.1 (11.8 –16.3) | .012 |
| ATA/AF-related hospitalization | 77 (16.7) | 97 | 8.7 (7.0 –10.4) | 92 (20.0) | 130 | 11.9 (9.9 –14.0) | .018 |
| ATA/AF-related ER visits | 42 (9.1) | 102 | 9.1 (7.4 –10.9) | 64 (13.9) | 135 | 12.4 (10.3 –14.5) | .02 |
| Repeat CA | 122 (26.5) | 195 | 17.5 (15.0 –19.9) | 115 (25.0) | 196 | 18.0 (15.5 –20.5) | .764 |
| **Male** | **Dronedarone  (*n*= 1115)^a^** | | | **Sotalol  (*n*= 1115)^a^** | | |  |
| Hospitalizations | 326 (29.2) | 600 | 22.5 (20.7–24.3) | 355 (31.8) | 662 | 24.8 (22.9–26.7) | .090 |
| ER visits | 428 (38.4) | 1368 | 51.4 (48.7–54.1) | 437 (39.2) | 1332 | 49.9 (47.2–52.6) | .444 |
| Outpatient office visits^b^ | 1099 (98.6) | 47559 | 1786.7 (1770.7–1802.8) | 1100 (98.7) | 45683 | 1711.5 (1695.8–1727.2) | <.001 |
| Other outpatient services^c^ | 1098 (98.5) | 28951 | 1087.7 (1075.1–1100.2) | 1090 (97.8) | 29573 | 1107.9 (1095.3–1120.6) | .026 |
| CV-related hospitalization | 157 (14.1) | 200 | 7.5 (6.5–8.6) | 197 (17.7) | 259 | 9.7 (8.5–10.9) | .007 |
| CV-related ER visits | 116 (10.4) | 206 | 7.7 (6.7–8.8) | 129 (11.6) | 210 | 7.9 (6.8–8.9) | .867 |
| ATA/AF-related hospitalization | 127 (11.4) | 154 | 5.8 (4.9–6.7) | 172 (15.4) | 217 | 8.1 (7.1–9.2) | .001 |
| ATA/AF-related ER visits | 102 (9.1) | 182 | 6.8 (5.8–7.8) | 103 (9.2) | 167 | 6.3 (5.3–7.2) | .407 |
| Repeat CA | 232 (20.8) | 357 | 13.4 (12.0–14.8) | 256 (23.0) | 401 | 15.0 (13.6–16.5) | .119 |
| ^a^Variables used in PSM are detailed in **Table S1**. HCRU was recorded at any time after index.  AF, atrial fibrillation; ATA/AF, atrial tachyarrhythmia/atrial fibrillation; CA, catheter ablation; CV, cardiovascular; ER, emergency room; HCRU, health care resource utilization; PSM, propensity score matching; PY, patient-year. | | | | | | | |

### Supplemental Table S7. HCRU prevalence rates in patients new to their index AAD (dronedarone or sotalol) among cohorts after PSM.

|  | **Dronedarone  (*n*= 549)^a^** | | | **Sotalol  (*n*= 549)^a^** | | |  |
| --- | --- | --- | --- | --- | --- | --- | --- |
| **Outcomes** | **Patients with event, *n* (%)** | **Total events** | **Rate per 100 PY (95% CI)** | **Patients with event, *n* (%)** | **Total events** | **Rate per 100 PY (95% CI)** | ***P* value** |
| **All-cause HCRU** |  |  |  |  |  |  |  |
| Hospitalization | 177 (32.2) | 310 | 23.4 | 242 (44.1) | 427 | 32.5 | <.0001 |
| ER visit | 235 (42.8) | 684 | 51.7 | 246 (44.8) | 842 | 64.0 | <.0001 |
| Outpatient office visit | 537 (97.8) | 24045 | 1817.1 | 544 (99.1) | 25093 | 1907.6 | <.0001 |
| Other outpatient services | 543 (98.9) | 15064 | 1138.4 | 535 (97.4) | 16324 | 1241.0 | <.0001 |
| **CV-related HCRU** | | | | | | | |
| Hospitalization | 96 (17.5) | 127 | 9.6 | 158 (28.8) | 203 | 15.4 | <.0001 |
| ER visit | 67 (12.2) | 129 | 9.8 | 89 (16.2) | 174 | 13.2 | .009 |
| Outpatient office visit | 509 (92.7) | 5187 | 392.0 | 521 (94.9) | 5543 | 421.4 | <.001 |
| Other outpatient services | 492 (89.6) | 4178 | 315.7 | 479 (87.2) | 3757 | 285.6 | <.0001 |
| Pacemaker implantation | 17 (3.1) | 20 | 1.5 | 35 (6.4) | 48 | 3.7 | <.001 |
| **ATA/AF-related HCRU** | | | | | | | |
| Hospitalization | 77 (14.0) | 97 | 7.3 | 148 (27.0) | 185 | 14.1 | <.0001 |
| ER visit | 60 (10.9) | 117 | 8.8 | 72 (13.1) | 146 | 11.1 | .067 |
| Outpatient office visit | 507 (92.3) | 4964 | 375.1 | 519 (94.5) | 5233 | 397.8 | .003 |
| Other outpatient services | 486 (88.5) | 3525 | 266.4 | 472 (86.0) | 3397 | 258.3 | .198 |
| Repeat CA | 156 (28.4) | 256 | 19.4 | 162 (29.5) | 275 | 20.9 | .371 |
| Prescription claims for AF-related drug | 549 (100.0) | 15285 | 1155.1 | 549 (100.0) | 16786 | 1276.1 | <.0001 |
| ^a^After PSM. Variables used in the PSM are detailed in **Table S1**. HCRU was recorded at any time after index.  AF, atrial fibrillation; CA, catheter ablation; ER, emergency room; HCRU, health care resource utilization; PSM, propensity score matching; PY, patient-year. | | | | | | | |

### Supplemental Figure S1. Study design schematic.


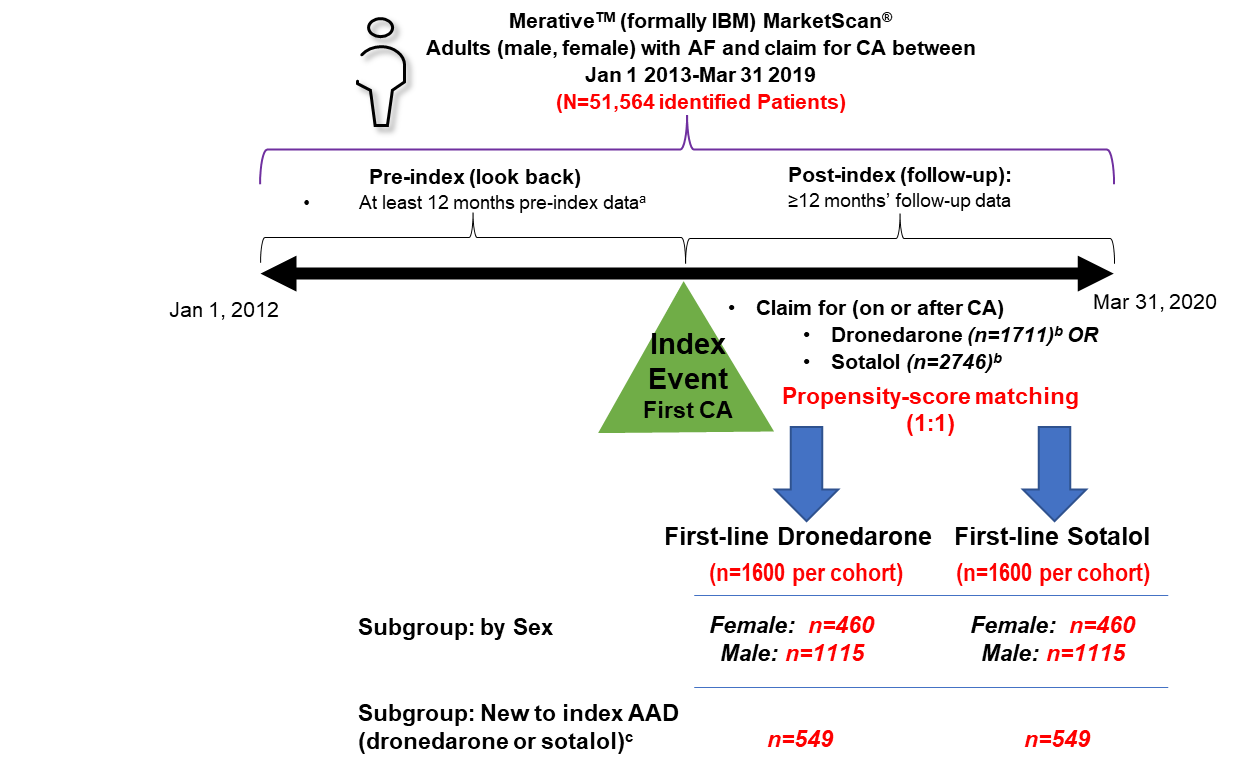


^a^Certain characteristics were assessed beyond 12-month baseline period.

^b^ Patient attrition is given in Figure 1.

^c^ With no use of index AAD (dronedarone or sotalol depending on treatment cohort) in 12 months before CA.

AAD, antiarrhythmic drugs; AF, atrial fibrillation; CA, catheter ablation.

### Supplementary Figure S2. Absolute standardized difference before and after PSM for each selected covariate


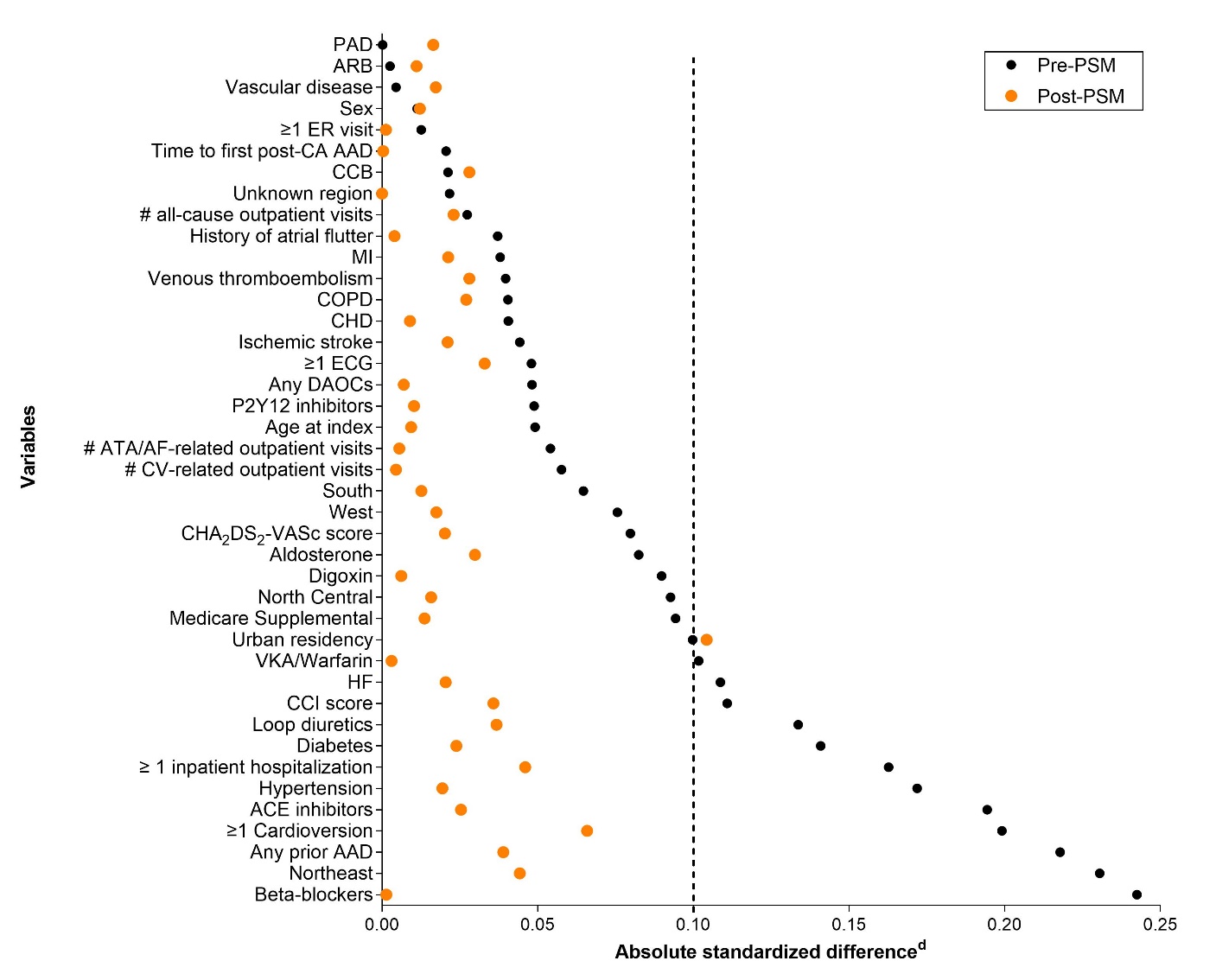


A covariate was considered balanced after PSM if absolute standardized difference (ASD) between cohorts was ≤0.1 (10%) (indicated by dashed line). Characteristics left unbalanced following PSM (ASD >0.1) were adjusted for as covariates.

AAD=antiarrhythmic drugs, ACE=angiotensin converting enzyme; ARB=angiotensin receptor blocker; CCB=calcium channel blocker; CCI= Charlson Comorbidity Index; CHD=coronary heart disease; COPD=chronic obstructive pulmonary disease; DOAC= direct acting oral anticoagulants; ECG= electrocardiogram; HF=heart failure; MI=myocardial infarction; PAD peripheral arterial disease; PSM=propensity score matching; VKA vitamin K antagonist.

### Supplemental Figure S3.Cumulative incidence rate for all-cause hospitalization, CV-related hospitalization, ATA/AF-related hospitalization, and repeat CA in (A) female and (B) male subgroups of patients with AF treated with dronedarone or sotalol post CA after PSM over 12-months follow-up


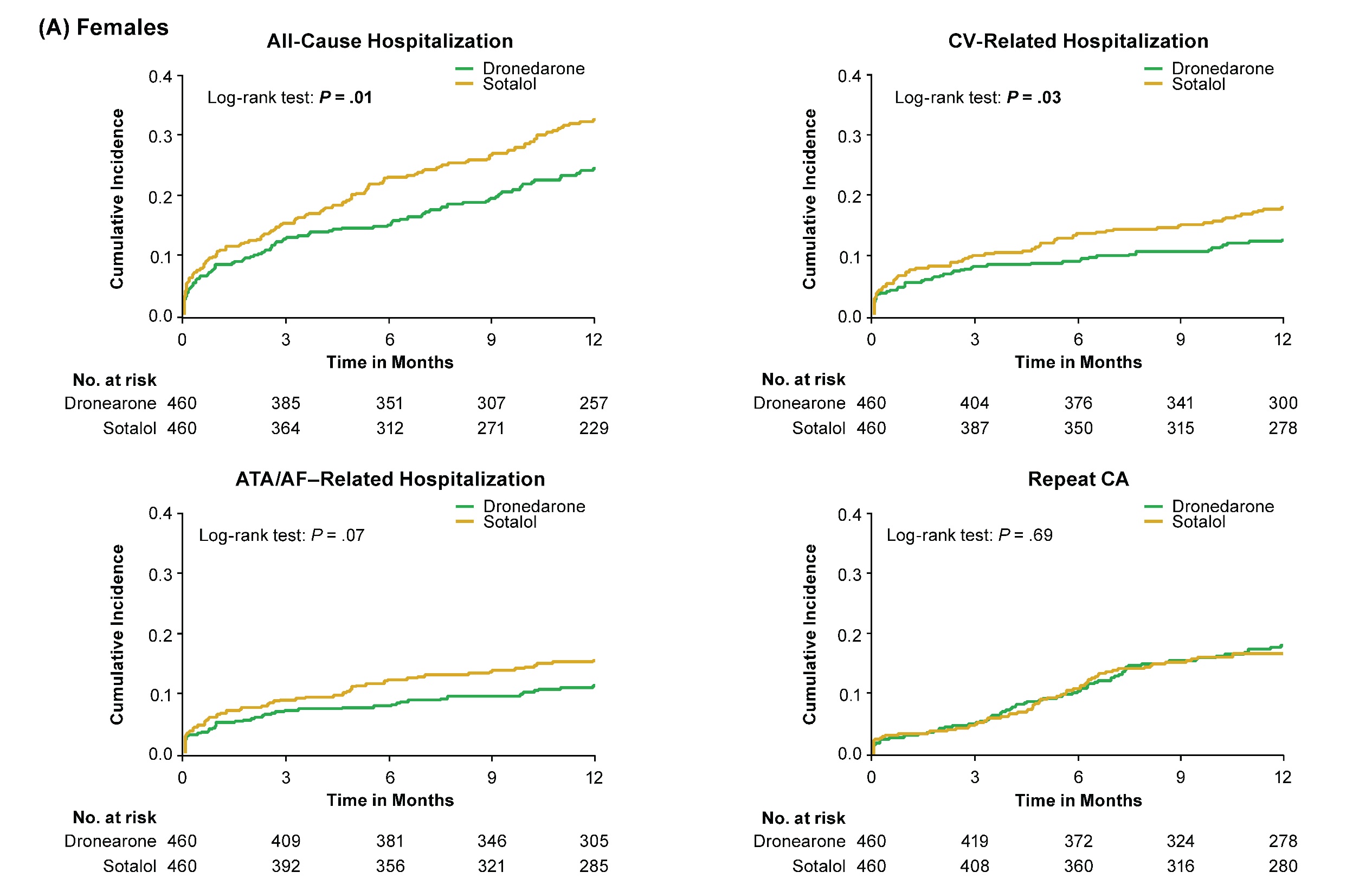


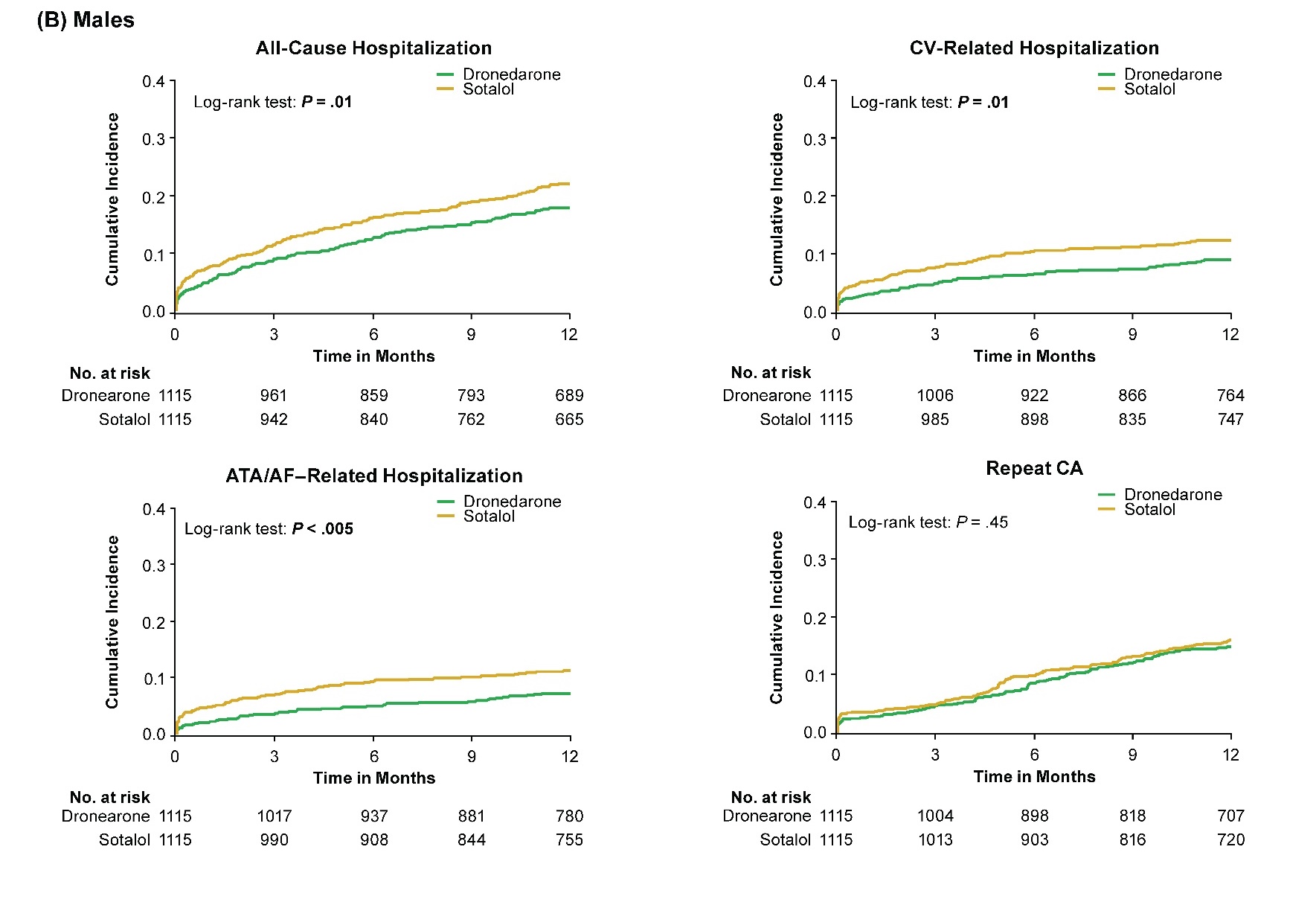


ATA/AF, atrial tachyarrhythmia/atrial fibrillation; CA, catheter ablation; CV, cardiovascular; PSM, propensity score matching. *p*-value comparing survival times between dronedarone and sotalol cohorts for each outcome.

### Supplementary Figure S4. Cumulative incidence rate for (A) CV-related hospitalization, (B) ATA/AF-related hospitalization, (C) pacemaker implantation, and (D) repeat CA for patients new to their index AAD (dronedarone or sotalol) in treatment cohorts after PSM over 12-months follow-up.


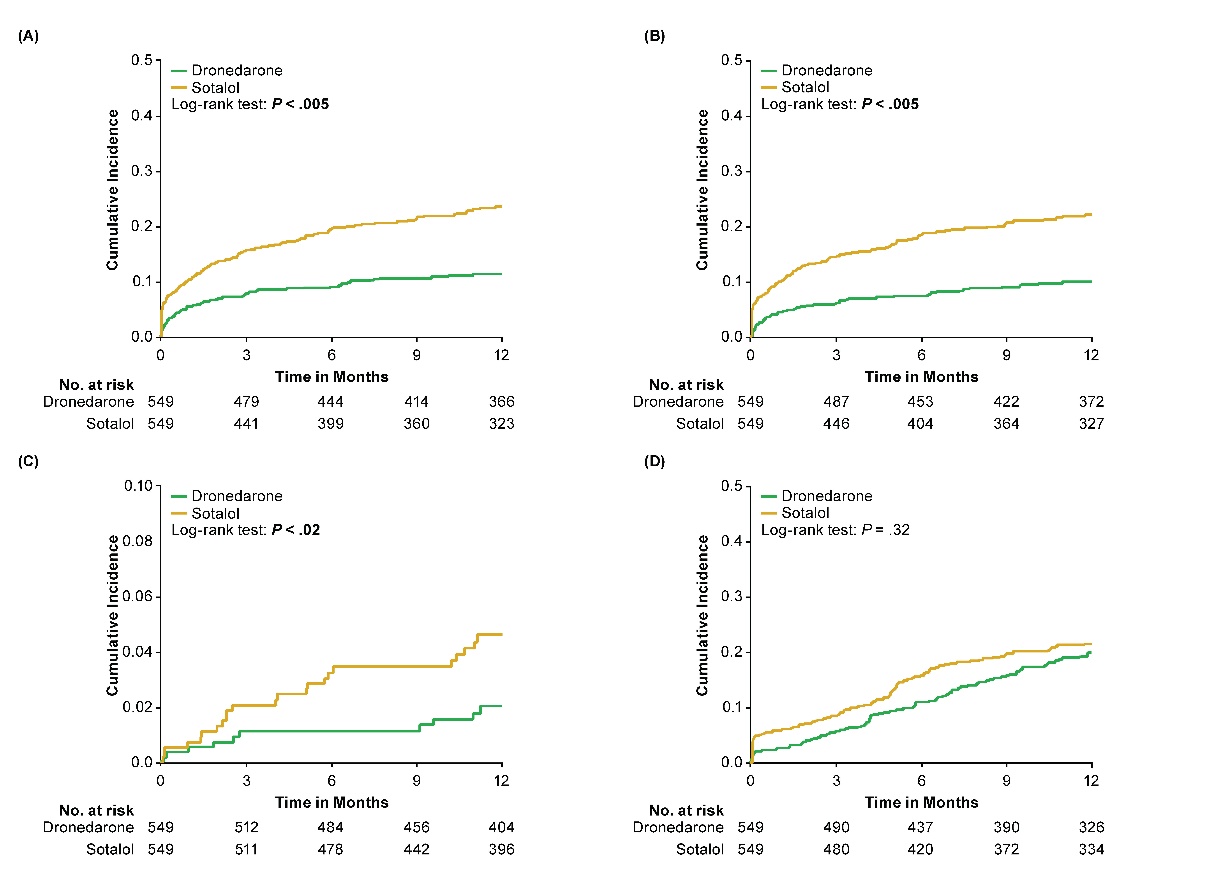


AAD, antiarrhythmic drugs; ATA/AF, atrial tachyarrhythmia/atrial fibrillation; CA, catheter ablation; CV, cardiovascular; PSM, propensity score matching. Patients new to index AAD had not received the study drug (dronedarone or sotalol prescribed as the first post-ablation AAD therapy) during the 12-month period prior to CA. *p*-value comparing survival times between dronedarone and sotalol cohorts for each outcome.
